# Identification and characterization of putative targets of VEGETATIVE1/FULc, a key regulator of development of the compound inflorescence in pea and related legumes

**DOI:** 10.3389/fpls.2022.765095

**Published:** 2022-09-21

**Authors:** Marcos Serra-Picó, Valérie Hecht, James L. Weller, Reyes Benlloch, Francisco Madueño

**Affiliations:** ^1^Instituto de Biología Molecular y Celular de Plantas, Consejo Superior de Investigaciones Científicas - Universidad Politécnica de Valencia, Campus Universidad Politécnica de Valencia, Valencia, Spain; ^2^School of Biological Sciences, University of Hobart, Hobart, TAS, Australia; ^3^Departamento de Biología Vegetal, Facultad de Farmacia, Universidad de Valencia, Valencia, Spain

**Keywords:** pea, legume yield, inflorescence architecture, legume compound inflorescence, secondary inflorescence meristem genes, *VEG1/FULc* gene, HUP54 gene

## Abstract

Inflorescence architecture contributes to essential plant traits. It determines plant shape, contributing to morphological diversity, and also determines the position and number of flowers and fruits produced by the plant, thus influencing seed yield. Most legumes have compound inflorescences, where flowers are produced in secondary inflorescences (I2), formed at the flanks of the main primary inflorescence (I1), in contrast to simple inflorescences of plants like Arabidopsis, in which flowers are directly formed on the I1. The pea *VEGETATIVE1/FULc* (*VEG1*) gene, and its homologs in other legumes, specify the formation of the I2 meristem, a function apparently restricted to legumes. To understand the control of I2 development, it is important to identify the genes working downstream of *VEG1*. In this study, we adopted a novel strategy to identify genes expressed in the I2 meristem, as potential regulatory targets of VEG1. To identify pea I2-meristem genes, we compared the transcriptomes of inflorescence apices from wild-type and mutants affected in I2 development, such as *proliferating inflorescence meristems* (*pim*, with more I2 meristems), and *veg1* and *vegetative2* (both without I2 meristems). Analysis of the differentially expressed genes using Arabidopsis genome databases combined with RT-qPCR expression analysis in pea allowed the selection of genes expressed in the pea inflorescence apex. *In situ* hybridization of four of these genes showed that all four genes are expressed in the I2 meristem, proving our approach to identify I2-meristem genes was successful. Finally, analysis by VIGS (virus-induced gene silencing) in pea identified one gene, *PsDAO1*, whose silencing leads to small plants, and another gene, *PsHUP54*, whose silencing leads to plants with very large stubs, meaning that this gene controls the activity of the I2 meristem. PsHUP54-*VIGS* plants are also large and, more importantly, produce large pods with almost double the seeds as the control. Our study shows a new useful strategy to isolate I2-meristem genes and identifies a novel gene, *PsHUP54*, which seems to be a promising tool to improve yield in pea and in other legumes.

## Introduction

The aerial organs of the plants derive from the shoot apical meristem (SAM). In annual angiosperms, the SAM goes through two developmental phases. During the vegetative phase, the SAM produces vegetative organs, leaves, and branches and, after the transition to the reproductive phase, the vegetative SAM is transformed into an inflorescence meristem that produces floral meristems that develop into flowers (Benlloch et al., 2007; Prusinkiewicz et al., 2007; Teo et al., 2013). Much of the huge diversity of plant forms depends on the wide variety in the architecture of the inflorescences (Weberling, 1992; Benlloch et al., 2007). Inflorescence architecture is important not only for its contribution to plant diversity but also because it regulates the production of flowers and fruits, thus having a great impact on crop yield (Park et al., 2014).

Legumes are the second most important crop, after cereals, with a world production of around 340 million tons per year (González-Bernal and Rubiales, 2016). Cereals surpass legumes in productive capacity; nevertheless, combining both crops brings notable advantages in total efficiency, as legumes strongly improve the access to nitrogen in the soil (Jensen et al., 2020; Rodriguez et al., 2021). Legumes are an essential source of nutrients in the human diet, and they are also of paramount importance for animal feed or forage. They are rich in protein (up to twice as high as cereals), fiber, unsaturated fatty acids, and carbohydrates (Iqbal et al., 2006; Beltrán and Cañas, 2018). Their nutritional properties make legumes a very healthy food highly recommended for human consumption. In addition, they compensate for some nutritional deficiencies in cereals, such as lysine and other valuable amino acids (Iqbal et al., 2006; Beltrán and Cañas, 2018).

**Figure 1:**
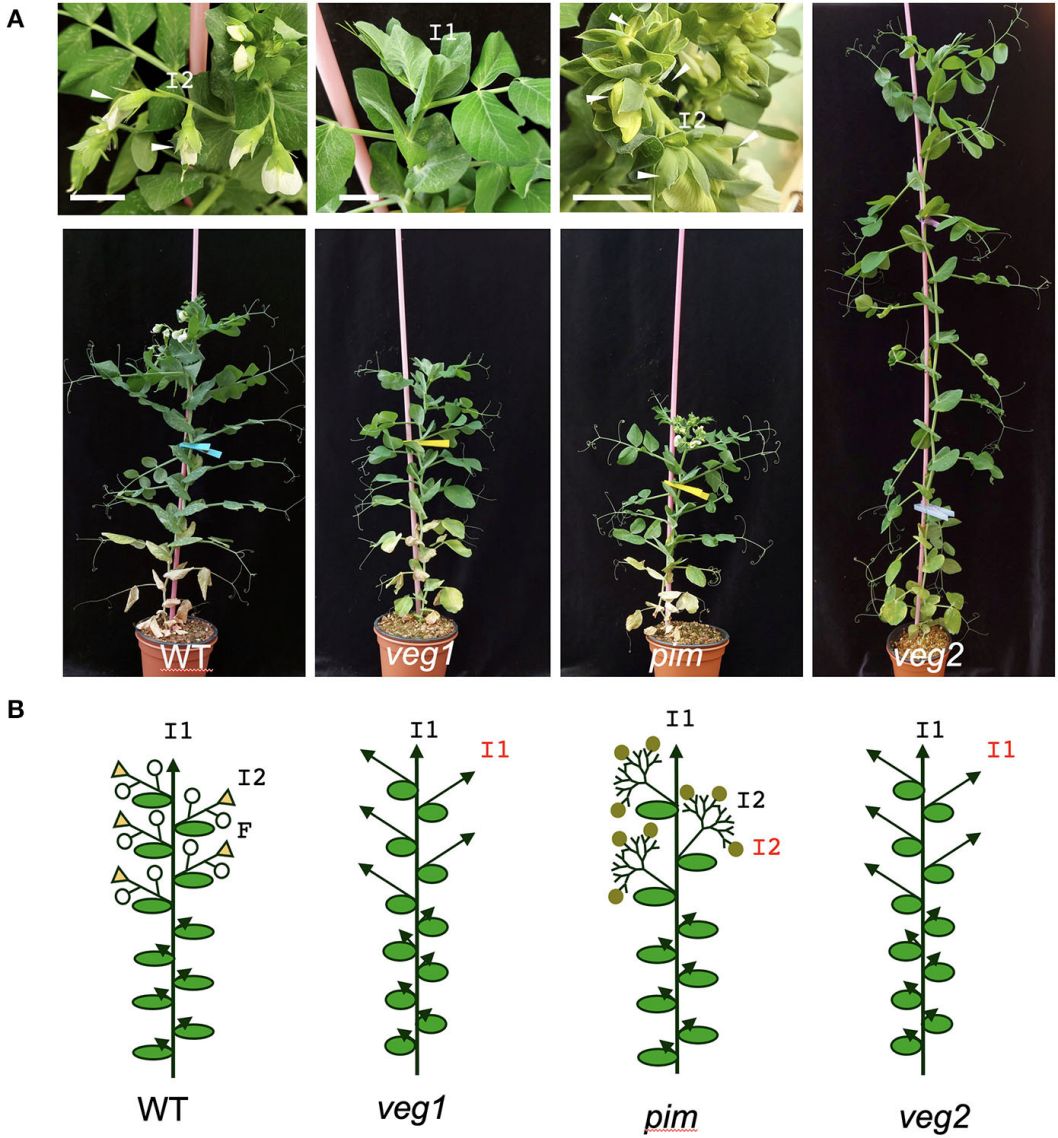
Inflorescence architecture of wild-type pea and *veg1, pim*, and *veg2* mutants **(A)** Images of wild-type (WT), *veg1, pim*, and *veg2* plants (lower panels). In the close-up pictures of the inflorescences of those plants (upper panels), I1 and I2s are marked. Flowers are marked with arrowheads. The WT usually has two flowers per I2, while in *pim* more flowers are produced. The *veg1* and *veg2* mutants neither produce I2s nor flowers. **(B)** Diagrams showing the inflorescence architecture of these genotypes. F, flower; white circles represent WT flowers; brown circles represent abnormal *pim* flowers; yellow triangles represent the stubs; scale bars: 2cm. The diagrams of the pea plants in **(B)** are much based on those published previously by us in this journal (Benlloch et al., 2015), with minor modifications.

In legumes, the most common inflorescence type is compound inflorescence (Weberling, 1989). In contrast to simple inflorescences, such as that observed in Arabidopsis, where flowers are directly formed by the SAM at the primary inflorescence stem (Benlloch et al., 2007), in compound inflorescences, the flowers are formed at the secondary or higher-order axes (Weberling, 1992; Benlloch et al., 2015). Thus, in legumes, the primary inflorescence (I1) meristem laterally forms secondary inflorescence (I2) meristems that produce the floral meristems (Figure 1). After producing a number of flowers, the I2 meristem terminates in the formation of a residual organ or stub (Figure 1; Benlloch et al., 2015).

Within legumes, the gene network controlling the identity of meristems in the inflorescence was first elucidated in pea (*Pisum sativum*) and possibly the one where it is best known (Berbel et al., 2012; Benlloch et al., 2015). Specification of inflorescence and floral meristem identity are governed by three types of genes. Pea I1 meristem identity is specified by *DETERMINATE*/*PsTFL1a* (*DET*), a homolog of the Arabidopsis *TERMINAL FLOWER 1* (*TFL1*) gene (Bradley et al., 1997; Foucher et al., 2003). As in the mutants of the Arabidopsis *TFL1* gene, development of the primary inflorescence (I1) meristem of pea *det* mutants is determinate, in contrast to wild-type pea, where development of the I1 meristem is indeterminate (Singer et al., 1999; Benlloch et al., 2015). *DET* function appears to be strongly conserved in legumes, and determinate mutants due to a mutation in *DET* homologs have been described in different legumes (Tian et al., 2010; Repinski et al., 2012; Dhanasekar and Reddy, 2015; Cheng et al., 2018). Pea floral meristem identity is mainly specified by *PROLIFERATING INFLORESCENCE MERISTEM* (*PIM*), homolog to the Arabidopsis *APETALA1* (*AP1*) gene (Taylor et al., 2002). *AP1* and *PIM* encode MADS domain transcription factors required for the formation of the floral meristem (Mandel et al., 1992; Berbel et al., 2001; Taylor et al., 2002). In *pim* mutant, the initiation of floral meristems from I2 meristems is impaired and as a result, the I2 meristems proliferate dramatically in an undifferentiated state and only eventually form some floral meristems (Taylor et al., 2002). Due to this proliferation, the inflorescence apices of *pim* mutants have more I2 meristems than the wild type (Figure 1B). Finally, the specification of I2 identity in pea depends on *VEGETATIVE 1/PsFULc* (*VEG1/PsFULc*), another MADS domain transcription factor gene from the same clade as Arabidopsis *AP1* and *FRUITFUL*, which is specifically expressed in the I2 meristem (Berbel et al., 2012). In *veg1* mutant plants, I2s are not formed and instead, they are replaced by vegetative I1 (vegetative) branches (Berbel et al., 2012). Mutants in the *VEGETATIVE2/PsFD* gene (*VEG2/PsFD*) exhibit an inflorescence phenotype that strongly resembles that of *veg1*, with defects in the formation of I2s, which are not produced in the plants of the *veg2-1* null mutant allele but instead are replaced by vegetative branches (Sussmilch et al., 2015). *VEG2* codes a bZIP transcription factor, homolog to Arabidopsis *FD*, which is required to upregulate *VEG1* upon floral transition (Abe et al., 2005; Sussmilch et al., 2015). This genetic network controlling the identity of the meristems in the inflorescence is also conserved in other legumes. For example, in *Medicago truncatula*, the whole network has been shown to work in the same way, with the *M. truncatula* homologs of the pea inflorescence genes, *MtFULc* (*VEG1/PsFULc*), *MtAP1* (*PIM*), and *MtTFL1* (*DET/PsTFL1a*), playing the same role in the inflorescence meristem identity genetic network than the pea genes (Cheng et al., 2018). Moreover, in soybean, *Dt1*, a homolog of pea *DET/PsTFL1a* that controls the determination of the I1 stem and *Det2*, an ortholog of *VEG1/PsFULc*, has also been isolated and characterized (Tian et al., 2010; Ping et al., 2014).

The formation of the I2 meristem, specified by *VEG1*, is a key step in the development of the legume compound inflorescence, and functional homologs of *VEG1* have only been described in legumes, which possibly reflects the fact that the function of this gene is most likely unique to the compound inflorescence of legumes. The activity of the I2 meristem determines the number of flowers that it produces. The number of flowers in the I2 is characteristic of each legume species and variety and influences the number of pods and, therefore, crop yield (French, 1990; Rubio et al., 2004).

Very little is known about how *VEG1* controls the formation of the I2s. The goal of this study is to identify genes expressed in the I2 meristem that might play a role in its development as targets of VEG1. With that aim, we have taken advantage of some of the molecular genetic tools available in pea: mutant lines in inflorescence meristem genes, as biological material (Benlloch et al., 2015), transcriptome and genome sequences, to analyze the transcriptome of these mutants (Alves-Carvalho et al., 2015; Kreplak et al., 2019), and virus-induced gene silencing (VIGS), as a method to study the function of the selected candidate genes (Constantin et al., 2004). In this study, we have compared the transcriptome in developing inflorescence tissue of the wild type, and the *veg1, pim*, and *veg2* mutants. That has led us to the identification of five genes expressed in meristems of the inflorescence apex. Finally, the silencing of these genes by VIGS showed that two of them control plant development and that one of these genes, *PsHUP54*, contributes to controlling the activity of the I2 meristem, and its silencing increases plant size and seed production.

## Materials and methods

### Plant materials and growth conditions

Wild-type (NGB5839 and Boneville) and mutant pea plants (*veg1/psfulc-1, pim-2*, and *veg2-1*) were selected for the present study. The original mutants, *psfulc-1, pim-2*, and *veg2-1*, have been previously described (Gottschalk, 1979; Murfet and Reid, 1993; Taylor et al., 2002). These mutations were backcrossed several times in the dwarf NGB5839 line (Hecht et al., 2007), so that the different mutations used in the study were in a genetic background as similar as possible. Plants were grown in a greenhouse at 21°C day temperature and 16°C night temperature and under a long-day (LD) photoperiod (16-h light/8-h darkness). When LD photoperiod conditions were required to be maintained, natural light was supplemented with lighting [400 W Phillips HDK/400 HPI (R)(N)]. Plants were irrigated periodically using Hoagland N°1 solution supplemented with oligoelements.

### Transcriptome analysis

For RNA-seq experiments, inflorescence apex samples (three biological replicates), from pea wild type, NGB5839 line, and *veg1, pim*, and *veg2* mutant plants were collected at floral transition, when the primary stem plant had formed 10 nodes (~4 weeks after germination). Each biological replicate consisted of 3–4 inflorescence apices (20–30 mg). Total RNA was extracted using the RNeasy Mini Kit (Qiagen) and treated with DNaseI (Turbo DNA-free kit INVITROGEN; Ref-AM1907), following the manufacturer's instructions. The quality of the RNA was checked on an Agilent 2100 Bioanalyzer instrument using the RNA6000 nano kit; the RIN values of the samples were between 9.1 and 10. To reduce the ribosomal RNA, polyA+ selection was used. Strand-specific RNA libraries were constructed using the TruSeq stranded mRNA kit (Illumina). Libraries were sequenced in a HiSeq2500 platform (Illumina) to produce 50-nucleotide single-end reads. Library construction and sequencing were performed at the genomics core facility at the Center for Genomic Regulation, Barcelona, Spain. Raw sequencing data have been deposited in GEO/NCBI (accession GSE188301).

The total number of reads in the samples varied between 18,292,370 and 22,907,817 (Supplementary Figure 1A). For RNA-seq data analyses, ribosomal RNA sequences were filtered out using SortMeRNA (Kopylova et al., 2012). Sequences of adapters were trimmed from the remaining reads using Trimmomatic (Bolger et al., 2014). The trimmed sequences were then aligned against the Pea transcriptome (PsUniLowCopy database, Ps Cameor database) using STAR (Dobin et al., 2013), and reads were counted with HTSeqCount (Anders et al., 2015). The number of reads mapped to the transcriptome is indicated in Supplementary Figure 1A. Analysis with a sample correlation matrix showed that the replicate libraries matched well and that the transcriptome of the different genotypes is clearly separated (Supplementary Figure 1B). DESeq2 with default parameters was used to perform differential expression analysis (Love et al., 2014). The identification of genes with opposite expression patterns was performed by constructing Venn Diagrams with the online tool Venny (Oliveros, 2007–2015). Genes with opposite expression patterns in *veg1* and *pim* samples were visualized in a heatmap created with the tool ClustVis (Metsalu and Vilo, 2015).

The RNA-seq data were validated by RT-qPCR gene expression analysis of selected genes in the wild type and mutants. For that, wild-type, *veg1*, and *pim* plants were grown for ~4 weeks, to node 10, as described above. The samples from inflorescence apices were collected, and RNA extraction and cDNA synthesis were done as described in the RT-qPCR section.

### Gene ontology (GO) term analysis

The analysis of the enrichment in gene ontology terms corresponding to *VEG1, PIM*, and *VEG2* differentially expressed genes was performed using the online tool AGRI-GO http://bioinfo.cau.edu.cn/agriGO/ (Du et al., 2010; Tian et al., 2017). For each pea transcript, we identified the best Arabidopsis homolog by using Basic Local Alignment Search Tool (BLAST). Then, we applied the singular enrichment analysis (SEA) for the identification of corresponding GO terms that were statistically overrepresented for each DEG list (*P* < 0.05).

### Characterization of gene expression levels by RT-qPCR

Total RNA was extracted with the SV Total RNA Isolation System (Promega) according to the manufacturer's instructions. RNA concentration of the samples was determined by spectrophotometer analysis using a NanoDrop 8000 (Thermo Scientific). Reverse transcription (RT) was conducted in a final volume of 20 μl and using 1 μg of total RNA as a template (MMLV high-performance reverse transcriptase, Epicenter) according to the manufacturer's instructions. The amplification efficiency was determined for all primer pairs used in RT-qPCR (Supplementary Table 1), and only primer pairs whose efficiency ranged from 90 to 100% were used. RT-qPCR reactions were performed in “*MicroAmp Optical 96-Well Reaction Plates with Barcode*” (Applied Biosystems) using the “*Premix PyroTaq Eva Green qPCR Mix Plus*” (GMC) kit, in a “*QuantStudioTM 3–96-Well 0.1 mL Block*” thermocycler (Applied Biosystems). RT-negative controls were maintained to monitor sample contamination with genomic DNA. As the reference and interplate calibrator, the pea gene *Actin11* was used, which has been validated in previously published RT-qPCR experiments in pea (Weller et al., 2009; Berbel et al., 2012). For each time point and/or tissue, three biological replicates were analyzed, and the results are presented as the average +/– standard deviation. Statistical significance was tested by a one-way ANOVA test, followed by Dunnett's multiple comparison test (1–4 asterisks indicating *P* < 0.05, < 0.01, < 0.001, or < 0.0001, respectively). Relative transcript levels were calculated following the Delta-Delta CT method (Livak and Schmittgen, 2001), using pea actin (PEAc14; ACCESSION U76193) as the reference gene. The primers used for PCR and RT-qPCR are detailed in Supplementary Table 1.

### Histological sections and *in situ* hybridization

**Figure 2:**
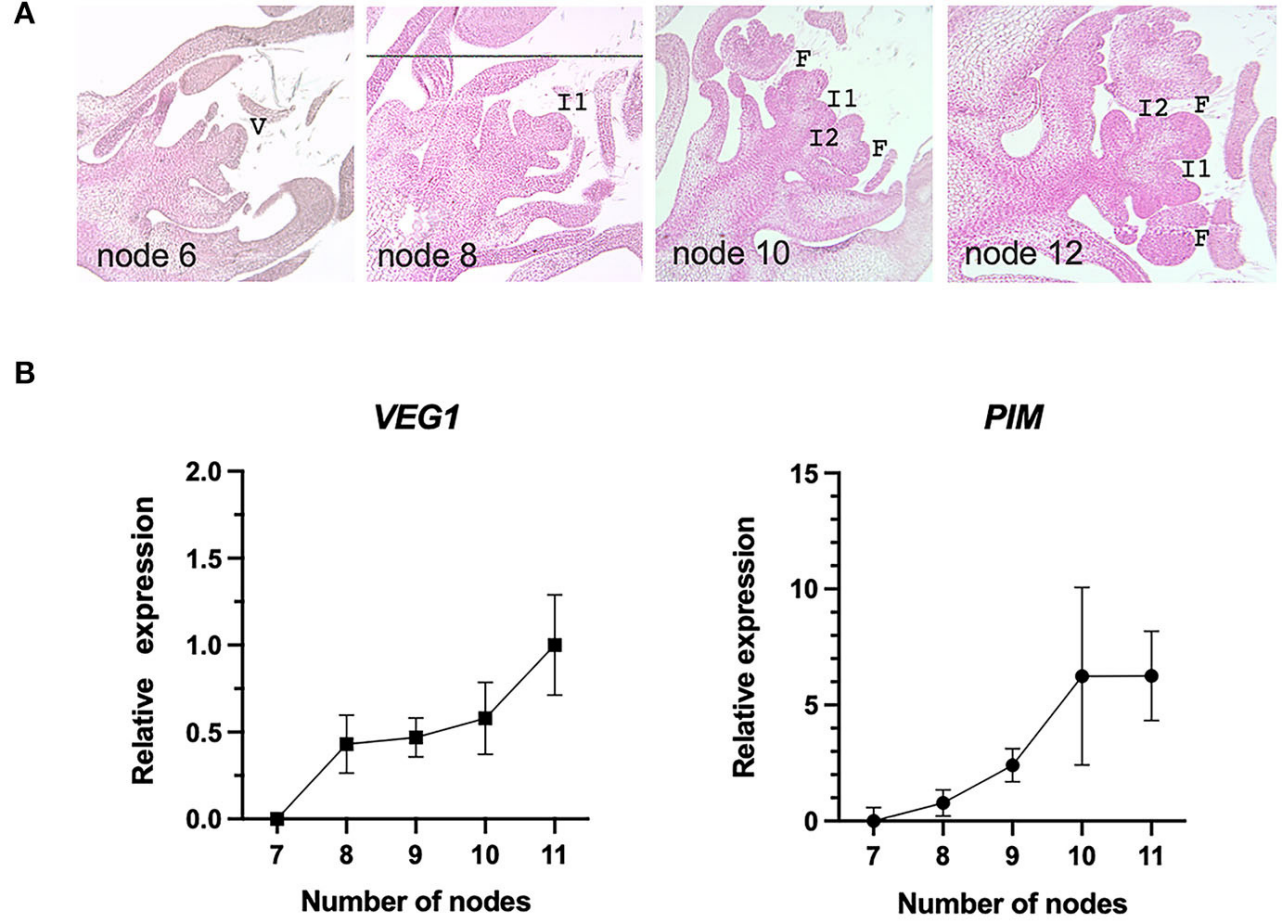
Floral transition in the NGB5839 pea line. **(A)** Histological sections of shoot apices of NGB5839 pea plants that had produced 6, 8, 10, or 12 nodes. I2 and floral meristems are observed from 10-node plants. **(B)** Relative expression levels of *VEG1* (left) and *PIM* (right), determined by RT-qPCR, of shoot apex samples collected from plants that had produced 7, 8, 9,10, or 11 nodes. Error bars correspond to standard deviation. V, shoot vegetative meristem; I1, primary inflorescence meristem; I2, secondary inflorescence meristem; F, floral meristem.

The histological study of floral transition and the *in situ* hybridization experiments (Figures 2, **6**) were done with 8-μm-thick longitudinal sections of inflorescence apices embedded in paraffin. For fixation, inflorescence apices were submerged in FAE solution (50% ethanol, 3.7% (v/v) formaldehyde, and 5% glacial acetic acid) and subjected to three vacuum pulses, according to the method described previously (Ferrándiz et al., 2000). FAE was replaced with a fresh solution and incubated for 2 h at room temperature. After fixation, samples were washed several times with 70% ethanol and afterwards stored in 70% ethanol. Paraffin embedding of the samples was performed in an automated tissue processor (LEICA TP 1020). Paraffin blocks were mounted using a LEICA EG1150H embedding device. A LEICA RM-2005 microtome was used to obtain 8-μm-thick sections. RNA *in situ* hybridization experiments were performed according to the methods previously described (Ferrándiz et al., 2000). For each gene, digoxigenin-labeled probes were generated using as a template a fragment of the coding sequence corresponding gene: PsCam039164 (350-bp fragment; positions 677–1,026), PsCam043276 (312-bp fragment; 39–350), PsCam043354 (350-bp fragment; positions 807–1,156), PsCam050808 (350-bp fragment; positions 744–1,093), and PsCam057706 (350-bp fragment; positions 1–350). Nucleotide positions are indicated using as reference the ATG codon. Each of the fragments was amplified by PCR using inflorescence apex cDNA as a template and cloned into the pGEM-T Easy vector (Promega) (for the primer sequences, see Supplementary Table 1). The gene fragments were selected in specific regions of the genes, with no significant sequence identity with related or distant genes, as indicated by BLAST analyses in the pea transcriptome. RNA anti-sense probes were generated with T7 RNA polymerase; sense probes were used as a control in each case, and they were generated using SP6 RNA polymerase.

### Virus-induced gene silencing

The gene fragments for VIGS constructs to downregulate the expression of *PsCam039164, PsCam043354, PsCam050808*, and *PsCam057706* genes were amplified from pea cDNA. The gene fragments corresponded to the same fragments used as probes for the *in situ* hybridization experiments and were highly specific, as mentioned above. The VIGS system combines two different plasmids: pCAPE1 and pCAPE2-PDS. These vectors contain, respectively, the RNA1 and RNA2 of the Pea early-browning virus (PEBV), under the control of the CaMV 35S promoter and the NOS terminator, in the binary vector pCAMBIA-1300 (Constantin et al., 2004). To generate the VIGS constructs, the vector pCAPE2-PDS was used, where PDS is flanked by several restriction enzymes that make it possible to replace the PDS fragment with the cDNA fragment of the gene to be silenced (Constantin et al., 2004). In all cases, NcoI and EcoRI restriction sites were used to subclone the gene fragments, with the exception of the *PsCam050808* gene, where NcoI and PstI sites were used. Gene fragments were PCR amplified as described in the previous section, using primers carrying the aforementioned restriction sites (Supplementary Table 1). The plasmid pCAPE2-Con, containing 400 bp of the GUS coding sequence, was used as VIGS negative control (Constantin et al., 2008).

The inoculation of plants was carried out as previously described in Constantin et al. (2004) with minor modifications as follows. For each experiment, 20–25 plants of the pea Boneville cv., about 3 weeks old when they have produced five leaves, were infiltrated with two *Agrobacterium tumefaciens* strains carrying the pCAPE1 and the pCAPE2 plasmids. Infiltrated plants were decapitated 5 days after inoculation, and in each plant, a single, basal, axillary shoot was kept and allowed to form a new primary shoot. These newly formed shoots were characterized as described below. As a positive control, 10 plants were inoculated with pCAPE2-PDS. Silencing of *PDS* (coding for pea PHYTOENE DESATURASE) leads to white leaves due to a lack of carotenoids and chlorophyll photooxidation (Constantin et al., 2008).

### Plant phenotypic characterization

For phenotypic characterization of VIGS plants, we scored a set of phenotypical traits: number and nodal position of secondary inflorescences, length of the main shoot internodes, number and complexity of leaves, length and structure of the secondary inflorescences (I2s) length of the stub, and length of the floral pedicels. For stub length, the ones that were so small that were barely visible and not measurable were considered to be 1 mm long, and the rest of the stubs were measured accordingly.

To determine when to collect the shoot apices from the pea plants, we scored the number of nodes in the primary stem to the node with the first folded leaf. For analysis of flowering, we considered the “flowering node” the first node with an I2 structure that produced a flower.

For the statistical analysis of parameters of VIGS plants, all the data containing multiple variables were analyzed by one-way ANOVA with *post-hoc* HSD Tukey's test taking as a significant difference depending on Bonferroni and Holm multiple comparisons. The statistical significance calculations for variables with data from two groups were performed with a two-tailed Student's *t-*test. Differences in expression were considered significant (^*^) when *P* < 0.05 and highly significant (^**^) when *P* < 0.01.

### Multiple sequence alignment

Putative *PsHUB54* homolog genes in other species were identified by using the protein-protein Basic Local Alignment Search Tool (BLAST) BLASTp at https://blast.ncbi.nlm.nih.gov/Blast.cgi (Altschul et al., 1990). Sequences from *Arabidopsis thaliana* HUP54 (AT4G27450), *Glycine max*XP_003543254.1, *Medicago truncatula* XP_013464732.1, and *Cicer arietinum* XP_004487686.1 proteins were aligned using the MEGAX and applying the ClustalW algorithm.

## Results

### Determining timing of floral transition in the pea line NGB5839

In order to elucidate the molecular mechanisms involved in the development of the secondary inflorescences (I2) of pea, we aimed to investigate the action of the VEG1 transcription factor, which specifies the identity of the I2 meristems (Berbel et al., 2012), by identifying its target genes. For this purpose, we adopted a genetic approach in which we compared the transcriptomes of inflorescence apices of wild-type pea and mutants in which the formation of I2 meristems was affected: *veg1, pim*, and *veg2*. In wild-type pea plants, after the floral transition, the flowers arise from secondary inflorescences that develop from the primary inflorescence (I1; Figure 1, Benlloch et al., 2015). In the *veg1* mutant, plants fail to produce these I2s, which are replaced by I1s (Figure 1). Conversely, in *pim* mutant plants, the I2 meristems proliferate before producing some flowers, so that more I2 meristems are formed (Figure 1, Taylor et al., 2002). Finally, *veg2* mutant plants show a phenotype similar to that of *veg1*, with no I2s, although the molecular basis for the phenotype is different from that in *veg1*, reflecting a defect in *VEG1* induction rather than direct impairment of its function (Figure 1, Sussmilch et al., 2015).

To select the most suitable time to compare the transcriptomes of the pea inflorescence mutants, we determined the timing of the floral transition, when the I2 meristems are initiated. With that aim, we characterized, both at the morphological and molecular levels, the development of the inflorescence of the reference line NGB5839, the wild-type genetic background of the inflorescence mutants used in this work (Hecht et al., 2007). First, we analyzed a series of shoot apex samples at different developmental stages (including apices from plants where the primary shoot had formed 6, 8, 10, or 12 nodes). Shoot apices were dissected, and the histological sections were prepared to determine the first node at which I2 and floral meristems could be observed. The results revealed that I2 or floral meristems could be readily observed in plants that had formed 10 nodes, but were not apparent in plants that had formed only 8 nodes (Figure 2A). In shoot apices of these 8-node plants no I2 or floral meristems could be observed while, on the contrary, we could readily identify those structures in plants that had formed 10 nodes (Figure 2A). These results indicated that these plants underwent floral transition between node 8 and node 10. Second, in the same type of samples, we used RT-qPCR to examine the expression of *VEG1* and *PIM*, I2 and floral meristem marker genes, respectively. Consistent with our previous observations, both genes displayed an increase in expression in the samples that had formed 8 and 10 nodes (Figure 2B). From these results, we decided to compare the transcriptome of apices of wild-type and mutant plants that had formed 10 nodes. In these plants, I2 meristems were visible, and *VEG1* expression was clearly detected, hence being an appropriate time to detect the expression of VEG1 regulatory targets.

### Transcriptome analysis of inflorescence apices of pea *veg1, pim*, and *veg2* mutants

**Figure 3:**
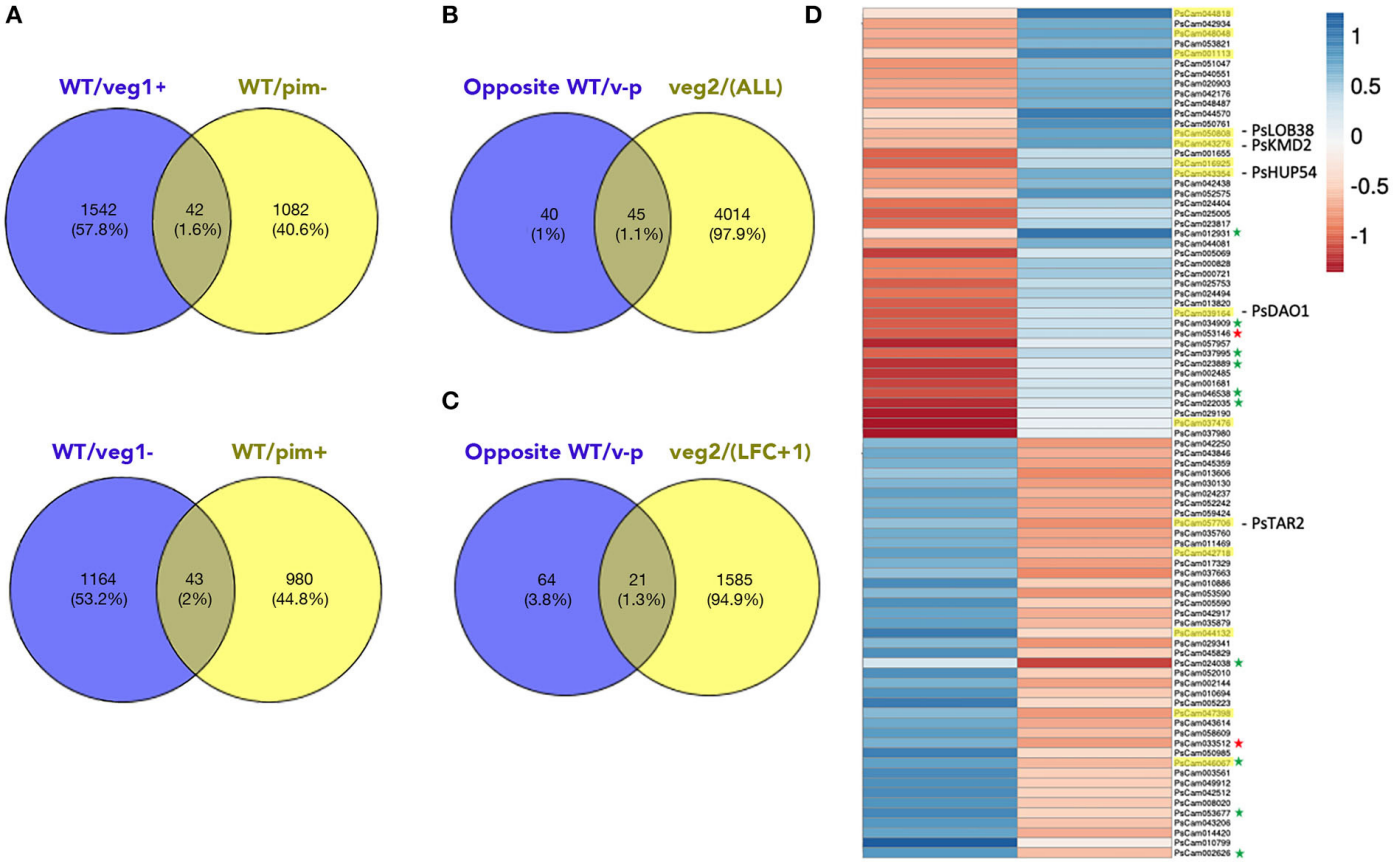
Transcriptome analysis of *veg1, pim*, and *veg2* apex samples compared to wild type. **(A)** Venn diagrams displaying genes with an opposite expression pattern in WT/*veg1* and WT/*pim* comparisons: 43 genes are downregulated in *veg1* (WT/veg1–) and upregulated in *pim* (WT/*pim*+) (lower panel); 42 genes are upregulated in *veg1* (WT/*veg1*+) and downregulated in *pim* (WT/*pim*-) (top panel). **(B)** Venn diagram identifying differentially expressed genes in WT/*veg2* among those with an opposite expression pattern in *veg1* and *pim* mutant backgrounds (opposite WT/v-p). **(C)** Venn diagram identifying differentially expressed genes in WT/*veg2* with a log of fold change >1 among those with an opposite expression pattern in *veg1* and *pim* mutant backgrounds (opposite to WT/v-p). **(D)** Heatmap displaying expression change of the 85 genes with an opposite expression pattern in WT/*veg1* and WT/*pim* (red shows downregulation of the gene in the mutant compared to the WT and blue indicates upregulation). Genes highlighted in yellow were selected for further characterization. Validation of the RNA-seq data was performed for 12 genes (marked with a star). We confirmed the opposite expression pattern in 10 out of 12 of those genes (green star). For two of the genes, the expression profile could not be confirmed (red star) (Supplementary Figure 2).

In order to identify the genes whose expression is associated with I2 meristems, inflorescence apex samples from wild-type, *veg1, pim*, and *veg2* plants were used to perform a transcriptome analysis by RNA-seq, comparing the transcriptome of each mutant to that of the wild-type line (WT). A comparison of the inflorescence apex transcriptome of *veg1* with that of the WT identified 2,792 differentially expressed genes (DEGs). Among those, 1,584 were upregulated and 1,208 were downregulated in *veg1* (Figure 3A, Supplementary Table 2). A similar comparison of WT and the *pim* mutant identified 2,148 DEGs (Figure 3A, Supplementary Table 2). Since *veg1* and *pim* have opposite phenotypes in terms of I2 meristem development, with *veg1* developing no I2 meristems and *pim* displaying a proliferation of I2 meristems, we identified which of the WT*/veg1* and WT*/pim* DEGs showed an opposite expression pattern. In this way, we found that 42 genes were upregulated in WT/*veg1* and downregulated in WT/*pim*, and 43 genes were downregulated in WT/*veg1* and upregulated in WT/*pim*, giving a total of 85 genes with an opposite expression pattern between WT*/veg1* and WT*/pim* (Figures 3A,D, Supplementary Table 3).

To validate the results of the RNA-seq, we randomly choose 12 genes among those 85 with opposite expression patterns and an LFC (log fold change) ≥1 for at least one of the transcriptomes (WT/*veg1* or WT/*pim*) and analyzed the expression of those genes by RT-qPCR in WT, *veg1*, and *pim* mutant apices (Supplementary Figure 2). We could confirm the results of the transcriptome analysis in 10 out of these 12 genes displaying a clear opposite expression pattern in *veg1* and *pim* mutant background compared to the wild type. Overall, these results indicate that our approach consisting of comparing transcriptomes of apex samples in these mutants was an effective method to identify the genes with an opposite expression pattern and possibly involved in VEG1-mediated control of I2 meristem development.

Finally, we characterized the transcriptome changes in the samples of the *veg2* mutant in comparison to WT. This comparison identified 4,059 DEGs, among which 2,163 were upregulated and 1,923 were downregulated in *veg2*. Both *veg1* and *veg2* mutants display a similar I2 phenotype (lacking I2 meristems), but the molecular bases for these phenotypes are different. Since we were interested in characterizing how VEG1 specifies the I2 meristem identity and controls its activity, we identified those genes with an opposite expression pattern in WT*/veg1* and WT*/pim* but that showed no expression change in *veg2* mutant (Figures 3B,C).

**Figure 4:**
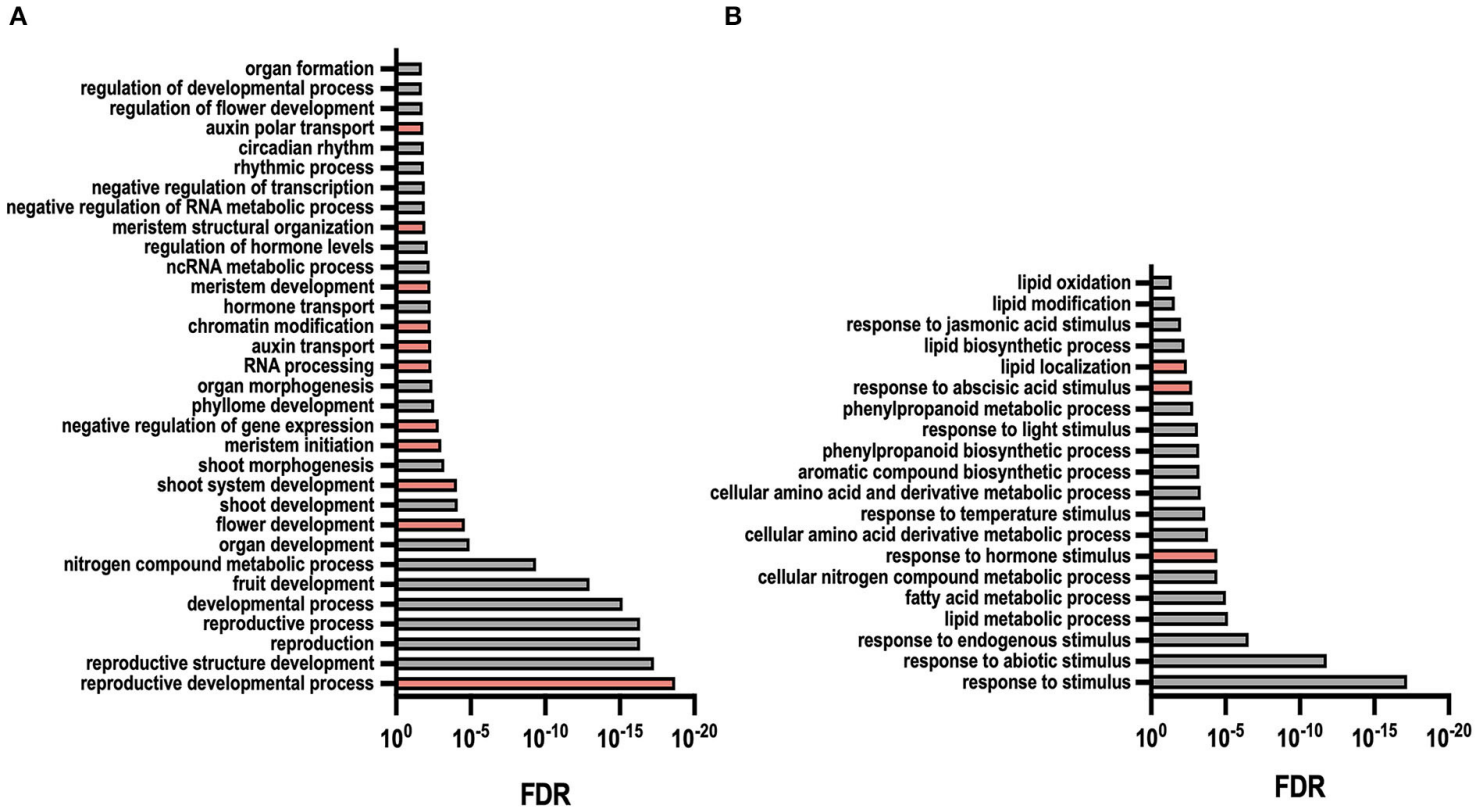
Gene ontology term (biological processes) enrichment among differentially expressed genes in WT/*veg1* transcriptome. **(A)** Selected GO terms returned from the analysis of genes upregulated in *veg1*. **(B)** Selected GO terms returned from the analysis of genes downregulated in *veg1*. Terms were selected according to their relation to previously described VEG1 function and/or potential VEG1 mechanisms of action. All depicted terms were overrepresented (false discovery rate (FDR) < 0.05). A complete list of all enriched GO terms for each analysis is detailed in Supplementary Table 4.

In order to identify the possible function of DEGs in each *transcriptomic* comparison (WT/*veg1*, WT/*pim*, and WT/*veg2*), we identified the putative homologs for each pea transcript by blasting their sequences against the Arabidopsis and *Medicago truncatula* databases. In the case of Arabidopsis, we identified close homologs for 74% of the pea transcripts, while in the case of Medicago, we could find the corresponding homologs for 77% of the pea transcripts. Because the information on genes was much more complete in this species, we used the Arabidopsis homolog genes to perform a gene ontology analysis (GO) with up- and downregulated genes and identified biological processes that were overrepresented in each of our datasets. GO term enrichment analysis with Arabidopsis homologs of WT/*veg1* upregulated pea transcripts returned enriched processes tightly related to reproduction, flower and meristem development, hormone transport, and regulation of transcription (Figure 4A, Supplementary Table 4), indicating that, as intended, we have identified genes that are involved in the reproductive development (as expected from a mutant such as *veg1*, showing impairment of I2 meristem initiation). Other biological processes that are enriched in this analysis include those referring to RNA metabolic processes (including non-coding RNA) and chromatin modification. Those terms, although less related to meristem development, point out to alternative mechanisms that could be contributing to the control of I2 meristem specification or activity. Analysis of GO term enrichment with Arabidopsis homologs of WT/*veg1* downregulated pea transcripts indicated processes related to the metabolism of different compounds, including lipids, amino acids, phenylpropanoids, or related to hormone response (more specifically, response to abscisic acid) (Figure 4B, Supplementary Table 4).

We performed a similar GO term enrichment analysis with DEGs identified in WT/*pim* and WT/*veg2* comparisons (Supplementary Table 4, Supplementary Figure 3). In both cases, enriched GO terms found with the upregulated genes indicated processes that are unequivocally related to reproduction, meristem initiation and development, and response to different environmental signals. In the case of WT/*pim*, among those terms, we found meristem and flower development, maintenance of meristem identity, floral organ development, or regulation of meristem growth. The negative regulation of developmental processes is another GO term indicating that we have identified genes related to the control of meristem specification and activity. A similar analysis with downregulated genes showed that terms, such as response to hormones (abscisic acid, ethylene, salicylic acid, and gibberellins), signal transduction, and transcription are significantly overrepresented in this dataset, in agreement with the loss of function of a transcription factor, such as PIM, controlling floral meristem specification (Supplementary Figure 3).

Finally, GO term enrichment with the WT/*veg2* dataset also identified significantly overrepresented processes, pointing out to an important environmental and hormonal regulation of meristem development (Supplementary Table 4, Supplementary Figure 3). On one hand, among the enriched terms identified with WT/*veg2* upregulated genes, we found an overrepresentation of terms related to meristem development (meristem initiation, meristem maintenance, and meristem growth), reproductive development (flower and fruit development), environmental signals (temperature), or hormone response. On the other hand, using the WT/*veg2* downregulated dataset, we find an overrepresentation of terms related to different biosynthetic processes (nitrogen compounds, protein and amines, and organic acids) (Supplementary Figure 3). This points to processes that control the metabolic status of the apices, as it was also observed in the enriched terms using the WT/*veg1* downregulated dataset. Interestingly, WT/*veg2* downregulated terms pointed out ribosome biogenesis and ribonucleoprotein complexes as very strongly enriched, which could indicate an unknown role of these processes in meristem regulation.

In order to narrow down our selection of genes putatively involved in the control of VEG1-mediated I2 meristem development, we applied several additional criteria to the list of 85 genes displaying opposite expression patterns between WT/*veg1* and WT/*pim*. Those criteria included *in-silico* analysis of gene expression pattern in different organs of pea plant (Pea gene expression atlas; Alves-Carvalho et al., 2015), function and expression pattern of the Arabidopsis homolog gene, contribution of these Arabidopsis homologs to relevant biological processes overrepresented in the GO analysis of WT/*veg1* DEGs, and whether the gene was up- or downregulated specifically in *veg1* (i.e., not differentially expressed in WT/*veg2*). With these criteria, we selected 14 genes for further functional characterization. As observed in the LFC (log fold change) (Table 1) and RPKM (reads per kilobase per million) data (Supplementary Table 5), though some of the selected genes did not comply with all criteria, all of them showed opposite regulation in *veg1* and *pim* inflorescences and met at least one other criterium.

**Table 1:** Genes selected for RT-qPCR and further expression analysis.

**Ps Cameor ID**	**LFC (WT/*veg1*)**	**LFC (WT/*pim*)**	**LFC (WT/*veg2*)**	**PsCam expression atlas^a^**	**Arabidopsis homolog**	**Arabidopsis protein and function**	**Arabidopsis GO-associated terms**
PsCam057706 (PsTAR2)	−0.3	0.4	–	Yes (2)^b^ LFPA	AT4G24670	Tryptophan aminotransferase involved in IAA biosynthesis	Indoleacetic acid biosynthetic^b^ process; flower development; maintenance of root meristem identity; shoot system development
PsCam050808 (PsLBD38)	0.5	−0.5	–	Yes (3) LFPA	AT3G49940	Transcription factor involved in anthocyanin biosynthesis and nitrogen availability signals	Regulation of gene expression
PsCam043354 (PsHUP54)	0.6	−0.6	0.6	Yes (1) LFPA	AT4G27450	Cellular response to hypoxia	–
PsCam039164 (PsDAO1)	2.5	−0.7	3.4	No PA	AT1G14130	IAA oxidase contributing to IAA degradation	Auxin homeostasis
PsCam043276 (PsKMD2)	0.5	−0.6	–	Yes (3) LFPA	AT1G15670	F-box protein involved in targeting type B-ARR proteins for degradation	Negative regulation of cytokinin
PsCam048048	0.3	−0.3	0.4	Yes (3) LFPA	AT2G36490	Repressor of transcriptional gene silencing	Nucleus; chromatin silencing
PsCam047398	−0.8	1.0	1.6	Yes (3) PA	AT5G59310	Lipid transfer protein	Response to abscisic acid ^c^
PsCam046067	−1.0	0.8	−1.3	Yes (2) LFPA	AT3G14160	2-oxoglutarate-dependent dioxygenase protein	Nucleus; oxidative DNA demethylase activity
PsCam044818	0.2	−0.7	–	Yes (2) LFPA	AT1G01040	RNA helicase involved in microRNA processing (dicer-like1)	Nucleus; DNA binding; flower development; vegetative to reproductive phase transition of meristem; RNA processing
PsCam044132	−0.6	0.3	−0.4	Yes (0) FPA	AT1G02205	–	–
PsCam042718	−0.3	0.3	–	Yes (2) 7476LFPA	AT1G69040	ACT-Domain protein involved in feedback regulation of amino acid metabolism	–
PsCam037476	10.7	−0.3	–	Yes (3) LFPA	AT3G02300	Regulator of chromosome condensation	–
PsCam016925	0.6	−0.2	–	Yes (0) LFPA	AT3G29075	Glycine-Rich protein	–
PsCam001113	0.3	−0.6	–	Yes (2) LFPA	AT2G19810	Oxidation-Related zinc-finger 1 involved in oxidative stress	Nucleus; chromatin silencing

a Expression according to the Pea gene expression atlas-−0, expression in shoot apices, NPKM value higher or close to 40; 1, NPKM value higher or close to 20; 2, NPKM value higher or close to 10; 3, NPKM value lower than 10. L, expression in leaves; F, expression in flowers; P, expression in pods; expression in other organs (different to leaves, flowers, pods, or shoot apices).

b Green font represents go-terms associated to genes upregulated in WT/veg1.

c Red font represents go-terms associated with genes downregulated in WT/veg1.

The GO term enrichment analysis suggested that several hormones are likely to play an important role in meristem initiation and development during floral transition in pea. In particular, auxin homeostasis and response to abscisic acid were the enriched terms in up- and downregulated gene sets, respectively (Figure 4). Accordingly, we selected three genes: PsCam039164, homolog of the *DIOXYGENASE FOR AUXIN OXIDATION 1* (*DAO1*) gene in Arabidopsis, involved in auxin degradation (Porco et al., 2016; Zhang et al., 2016), hereafter named *PsDAO1*; Pscam057706, corresponding to the pea *PsTAR2* gene, homolog of the Arabidopsis *TRYPTOPHAN AMINOTRANSFERASE RELATED 2* (*TAR2*) gene, both of them involved in auxin biosynthesis (Stepanova et al., 2008; Tivendale et al., 2012; McAdam et al., 2017); and PsCam047398, homolog to an Arabidopsis lipid transfer protein strongly upregulated by abscisic acid (Gao et al., 2016). *PsTAR2* was of particular interest, since the expression of its Arabidopsis ortholog *TAR2* increases strongly at the shoot apical meristem upon floral induction, and its expression at the SAM was restricted to the peripheral zone, where lateral organs are initiated (ePlant: https://bar.utoronto.ca/eplant/; Waese et al., 2017). The selection of *PsDAO1* as a candidate was supported as well by a very discrete expression pattern of its Arabidopsis homolog in the rib meristem at the SAM (ePlant). Finally, PsCam047398, besides being upregulated by ABA, is related to lipid transport and lipid localization, biosynthesis, and modification, which came up as enriched GO terms in our previous analysis, supporting the selection of this gene for further investigation. A fourth hormone-related selected gene was PsCam043276, a homolog of the *KISS ME DEADLY 2* (*KMD2*) gene of Arabidopsis, which, together with *KMD1*, is involved in cytokinin signaling and has been shown to have an impact on shoot apical meristem size when overexpressed (Kim et al., 2013).

PsCam050808, hereafter named *PsLBD38*, is a homolog of *LATERAL ORGAN BOUNDARIES 38* (*LBD38*), an Arabidopsis transcription factor involved in defining lateral organ boundaries that are repressed at the SAM upon floral induction and that has been related to the control of flowering time in rice plants (ePlant; Albinsky et al., 2010). PsCam043354 (hereafter *PsHUP54*) is a homolog of the *HYPOXIA REPSONSE UNKNOWN PROTEIN 54* (*HUP54*) for which very little functional information is available. In Arabidopsis, *HUP54* is transiently upregulated at the SAM during floral transition. Its expression at the SAM is restricted to the rib meristem and the peripheral zones, being quite low in the central zone (ePlant). The *HUP54* gene has been shown to be regulated in floral buds by SHINE transcription factors and gibberellins (Shi et al., 2011). From all these data, *PsLBD38* and *PsHUB54* were selected for further characterization.

Finally, we selected a number of genes that could eventually reveal a role for different mechanisms in the regulation of I2 meristem specification and activity: PsCam048048, PsCam46067, and PsCam001113 were selected based on the function of their Arabidopsis homolog genes in chromatin silencing (GO term enriched in the VEG1 upregulated dataset) and PsCam044818 based on the relation of its Arabidopsis homolog with RNA processing (RNA metabolic process including ncRNA) and to the vegetative to the reproductive phase transition. The Arabidopsis homologs of these genes are expressed in different domains of the SAM, and their expression level changes during the floral transition (ePlant). PsCam037476 was selected based on its strong upregulation in the *veg1* mutant, together with the fact that its homolog in Arabidopsis, belonging to the Regulator of Chromosome Condensation (RCC) protein family, displays a strong expression at the SAM (ePlant). PsCam044132, PsCam042718, and PsCam016925 were included in the selected list based on the expression changes of their corresponding homolog genes in Arabidopsis, which in all cases were upregulated in the SAM upon a floral transition (ePlant) and the last two were, in addition, VEG1 specific (not significant changes were detected in WT/*veg2* transcriptome).

### Expression analysis of genes differentially expressed in *veg1*

**Figure 5:**
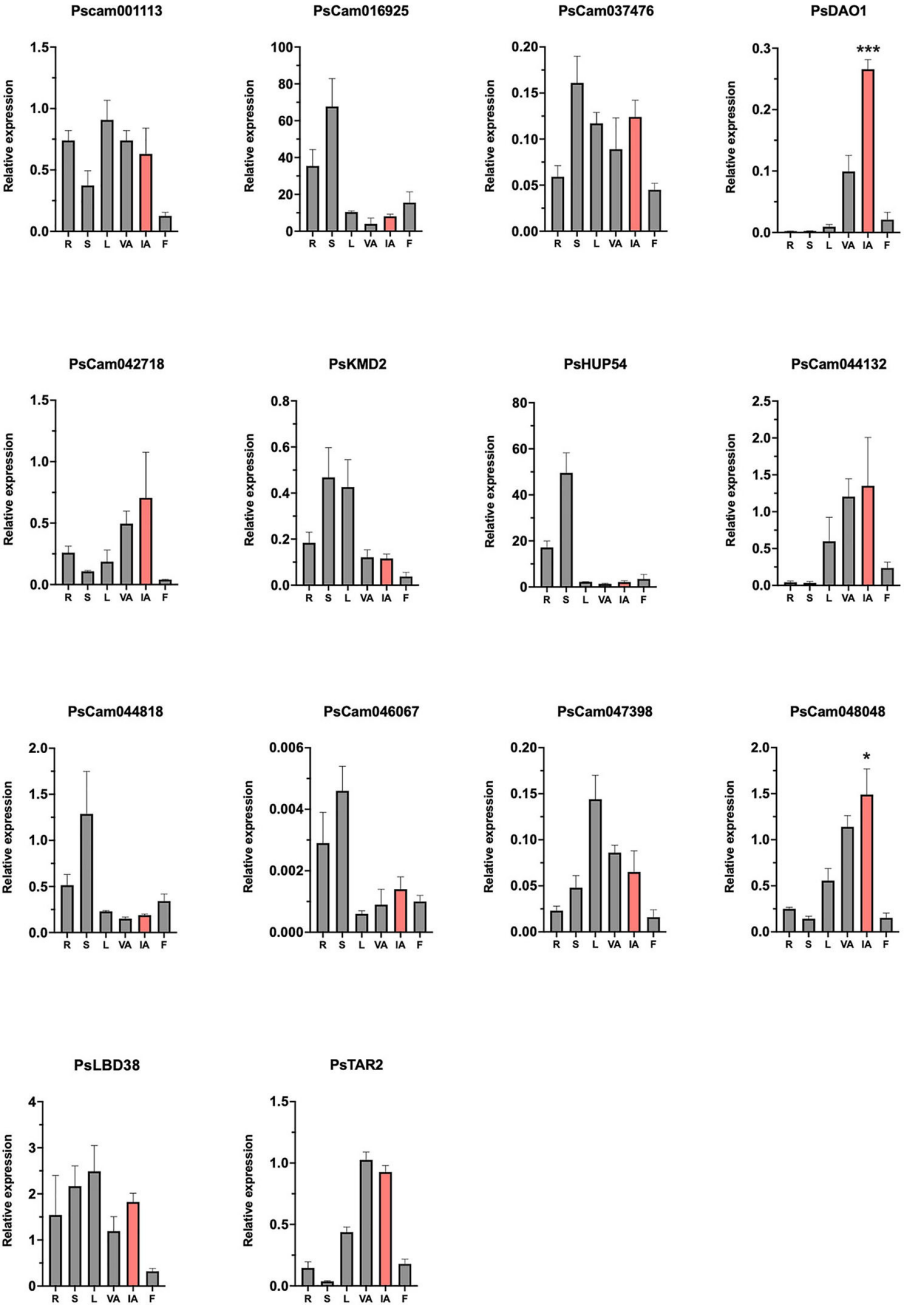
Expression analysis by RT-qPCR of candidates for VEG1 target genes in different pea plant organs. Relative mRNA levels were determined by RT-qPCR. For the analysis, different samples were collected from wild-type plants: roots (R), shoots (S), leaves (L), vegetative apices (VA), inflorescence apices (IA - highlighted in red), and flowers (F). Roots, shoots, leaves, and vegetative apices were collected from 3-week-old plants, before the floral transition; the inflorescence apices (highlighted in red) were collected from ~5-week-old plants after the floral transition had occurred, and flowers were collected at anthesis. Error bars correspond to standard deviation.

Since the functional characterization of pea genes is still challenging, we decided to narrow down the list by performing an initial gene expression analysis of the 14 selected genes to see whether they are expressed in the inflorescence apex, as expected for genes involved in I2 meristem development, and whether this expression is inflorescence specific. We analyzed the expression of these genes in different organs of pea wild-type plants, including roots, stem, leaves, vegetative apices, inflorescence apices, and flowers. The expression of the 14 genes was detected in the inflorescence apex at various levels (Figure 5). However, only in the case of *PsDAO1* and PsCam048048, higher expression in the inflorescence apex was statistically significant when compared to the vegetative apex. The expression of PsCam00113, PsCam037476, PsCam042718, PsCam044132, PsCam047398, *PsLBD38*, and *PsTAR2* was similar in inflorescence apices than in other organs, indicating that these genes could have a role in I2 meristem, but they would probably have additional roles in the development of other plant organs. We could distinguish the third group of genes, including PsCam016925, *PsKMD2, PsHUP54*, PsCam044818, and PsCam046067, in which we were able to detect expression in the inflorescence apex, but the level of expression was lower than in other organs of the plant. A low level of expression of these genes in the inflorescence apex did not discard them as candidates, since a restricted expression in specific regions of the apex (as in the case of I2 meristem with specific expression only) can be masked in RT-qPCR assays.

**Figure 6:**
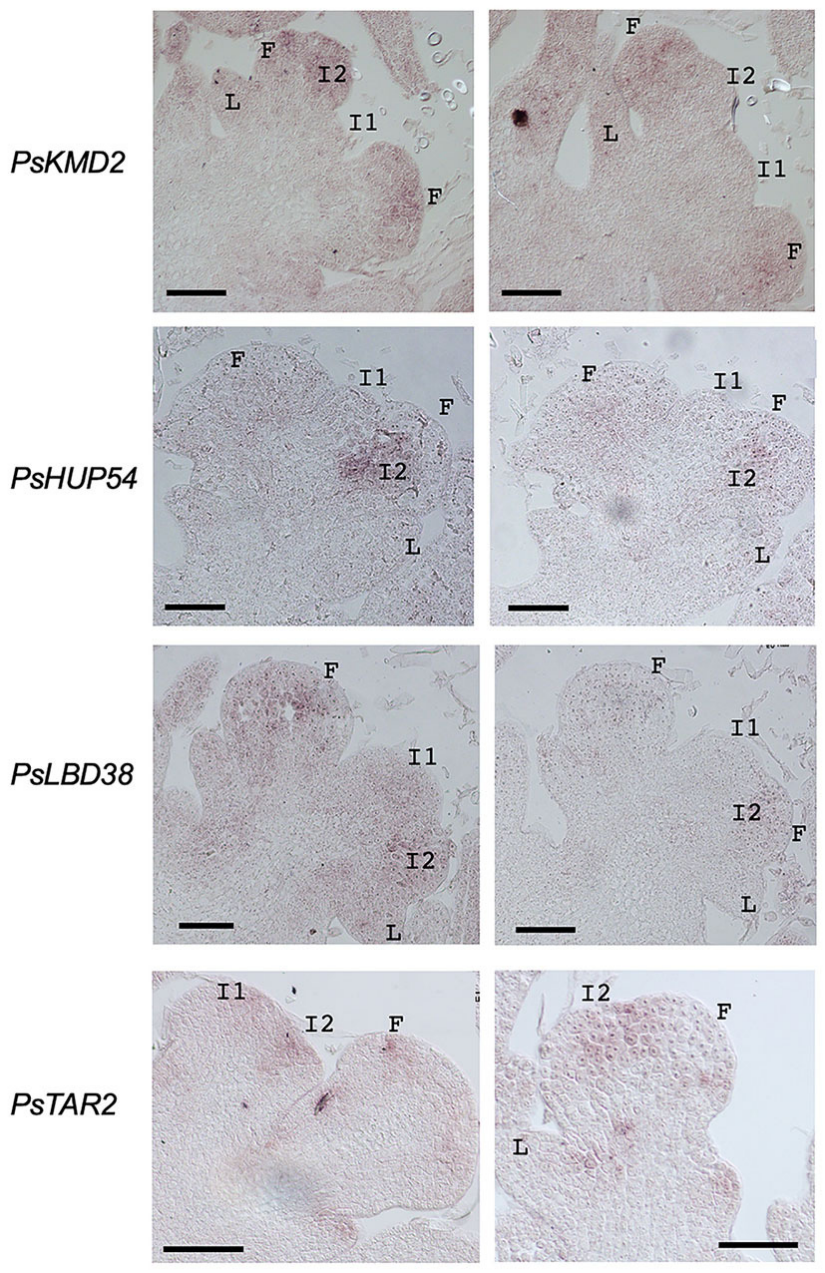
Expression analysis by *in situ* hybridization in pea inflorescence apices of selected candidates for VEG1 target genes. Sections of wild-type pea inflorescence apices were hybridized with anti-sense probes for the genes *PsKMD2, PsHUP54, PsLBD38*, and *PsTAR2*. L, leaf primordium; I1, primary inflorescence meristem; I2, secondary inflorescence meristem; F, floral meristem/primordium. Scale bars: 100 μm.

Considering the expression and the information on the corresponding Arabidopsis homologous genes, we decided to characterize the expression pattern by *in situ* hybridization in pea inflorescence apices of five of these candidates: *PsDAO1, PsKMD2, PsHUP54, PsLBD38*, and *PsTAR2* (Figure 6). The *in situ* hybridization experiments worked for four of the five genes, but not for *PsDAO1*. The probes of the four remaining genes showed hybridization in the meristems of the inflorescence apices (Figure 6), while no signal was detected in this tissue for any of these genes with the negative control sense probes (Supplementary Figure 4). *PsKMD2* and *PsLBD38* showed hybridization in both I2 and floral meristems, indicating that the expression in the inflorescence of these genes is not specific for the I2 meristem (Figure 6). In contrast, for *PsHUP54* and *PsTAR2*, the hybridization signal was apparently restricted to the I2 meristems, implying the possible function of these genes in the specification of I2 identity (Figure 6).

### Functional analysis by VIGS of selected gene points *PsHUP54*/PsCam043354 as possible regulators of I2 activity

In order to analyze the function of selected candidates, we carried out VIGS experiments in pea to silence the expression of four genes: *PsHUP54, PsTAR2, PsLBD38*, and *PsDAO1*. *PsHUP54* and *PsTAR2* were selected for functional analysis because they showed an expression profile apparently restricted to the I2 meristem (Figure 6). *PsLBD38* was also selected because, although it showed expression in both I2 and floral meristems, RT-qPCR detected a high level of expression in inflorescence apices (Figures 5, 6). Finally, we decided to also include *PsDAO1*, despite the fact that we could not detect its expression by *in situ* hybridization, due to its strong expression in inflorescence apices detected by RT-qPCR (Figure 5).

The VIGS constructs for these four genes were generated using the pCAPE2-PDS vector (Constantin et al., 2004). As a negative control, plants agroinfiltrated with a *GUS*-VIGS construct, containing the *Escherichia coli UidA* gene, were used (*GUS*; Jefferson et al., 1987). The pCAPE2-PDS construct (*PDS*-VIGS), inoculated in 10 plants, was used as a positive control. All the *PDS*-VIGS plants produced white leaves (Constantin et al., 2008), indicating that the silencing was highly efficient in our experiment. The effect on the wild-type plants of the VIGS constructs for the four different pea genes was studied by analyzing different parameters: length of the stem internode, the leaf, the I2 stem, the stub, and the floral pedicel, as well as the number of leaflets (Table 2). *PsTAR2-* and *PsLBD38*-VIGS plants did not show evident phenotypes, and the only apparent defect observed was in the length of the floral pedicels, which was significantly shorter in *PsLBD38*-VIGS plants than in the *GUS*-VIGS control plants (Supplementary Figure 5, Supplementary Table 6).

**Table 2:** Characterization of morphological alterations in *PSDAO1* and *PsHUP54*-VIGS plants.

**VIGS construct**	**Parameters**								
	**Internode length^b^ (cm)**	**Leaflet number^c^**	**Leaf length^c^ (cm)**	**I2 length^d^ (mm)**	**Stub length (mm)**	**Floral pedicel length (cm)**	**Pod size^e^ (mm)**	**Seed weigth^f^ (gr)**	**Seed no./pod**
GUS	2.25 ± 0.48	3.62 ± 0.72	6.53 ± 1.62	9.75 ± 3.45	1.52 ± 0.67	5.74 ± 0.94	56.93 ± 8.03	3.66 ± 0.19	2.53 ± 1.25
PsDAO1	**0.74 ±0.300^a^**	3.32 ± 0.98	**2.89 ±1.41^a^**	3.14 ± 1.53	1.77 ± 0.82	**4.45 ±1.15^a^**	N.D.	N.D.	N.D.
PsHUP54-m-s^g^	**3.15 ±0.87^a^**	3.75 ± 0.75	**9.19 ±2.04^a^**	**30.46 ±16.73^a^**	**5.97 ±6.07^a^**	**8.16 ±1.84^a^**	**67.31 ±8.65^a^**	3.55 ± 0.18	**4.33 ±1.97^a^**
PsHUP54-w^h^	**1.93 ±0.49**	3.40 ± 0.84	**4.81 ±1.30**	11.00 ± 6.69	**3.6 ±2.5**	5.33 ± 0.52			

The values correspond to mean ± standard deviation.

a The data in bold correspond to values with statistically significant variation with respect to the values of control GUS-VIGS plants. For statistical analysis, one-way ANOVA test with Bonferroni and Holm inference test was used.

b Values correspond to the average length of the internodes of the stem before the first reproductive node.

c Leaflet number and leaf length correspond to the leaves at the first reproductive node and at the previous one.

d Values correspond to the length of the “stem” of the I2s of each plant.

e The values correspond to the pods at the first three reproductive nodes. Seeds in those pods were used to estimate seed weight.

f Values for seed weight correspond to the average weight of five groups of 10 seeds from each plant.

n.d., not determined.

g Analyzed in PsHUP54-VIGS plants that showed moderate-strong phenotype, characterized for being very high plants > 15 cm (15 out of 19 plants in the experiment exhibited that moderate-strong phenotype).

h Analyzed in PsHUP54-VIGS plants that showed weak phenotype (4 out of 19 plants in the experiment exhibited a weak phenotype).

**Figure 7:**
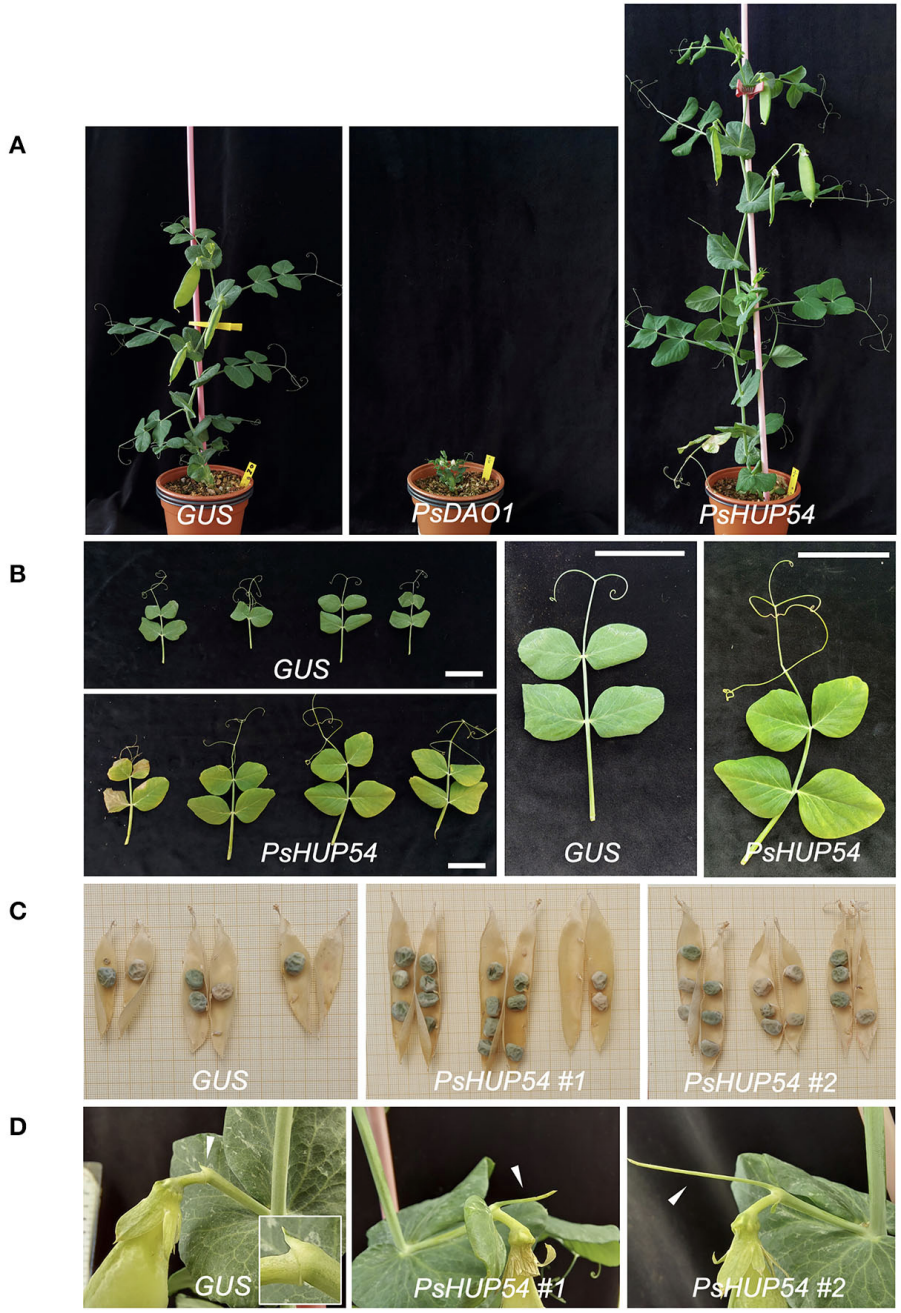
Phenotype of pea VIGS plants for genes *PsDAO* and *PsHUP54*. (**A)** Representative 10-week-old *GUS*-*VIGS* (control), *PsDAO1*-VIGS, and *PsHUP54*-VIGS plants. **(B)** Leaf defects of *PsHUP54*-VIGS. Leaves were sampled from the fourth node of different plants. *PsHUP54*-VIGS leaves are bigger and slightly lighter than *GUS*-VIGS leaves. **(C)** Seed number and pod defects of *PsHUP54*-VIGS plants. The images show the three first pods of different plants. **(D)** Increased stub length in *PsHUP54*-VIGS plants. Stubs are marked with arrowheads. Detail of the stub in a control *GUS*-VIGS plant is shown in a close-up. Scale bars: 5 cm.

In contrast, *PsDAO1-*VIGS plants and *PsHUP54*-VIGS plants consistently showed strong phenotypes when compared to *GUS*-VIGS control plants. In the case of *PsDAO1*-VIGS, 3 weeks after infiltration with the VIGS construct, plants displayed conspicuous necrosis in the first leaves not observed in plants infiltrated with the *GUS*-VIGS control construct (Supplementary Figure 6). Later on, once the *PsDAO1*-VIGS plants had grown for ~10 weeks, all plants were notably smaller than the *GUS*-VIGS plants (Figure 7A), and the internodes, leaves, and floral pedicels were significantly shorter than those of the *GUS*-VIGS plants (Figure 7B).

In contrast, *PsHUP54*-VIGS plants grown for a similar time period were notably bigger than *GUS*-VIGS plants overall (Figure 7A), with significantly larger I2 stems, stubs, and leaves, frequently yellowish (Figures 7B,D, Table 2). The phenotype was particularly striking in the case of the I2 stem and the stub, the residual organ formed by the I2 meristem once it stops producing floral meristems (Benlloch et al., 2015). Thus, in the moderate-strong phenotype *PsHUP54*-VIGS plants (big plants 15–40 cm high; 15 out of a total of 19 *PsHUP54*-VIGS plants), the I2 stem and the stub showed close to 3- to 4-fold increase in length relative to the control, respectively (Figure 7D, Table 2). In addition, in one of the *PsHUP54*-VIGS plants, the elongated I2 gave rise to an additional second flower. This group of plants also produced pods that were significantly larger and contained more seeds than those from the control plants, with no decrease in seed weight (Figure 7C, Table 2). The remaining four *PsHUP54*-VIGS plants looked quite similar to the control plants. The only defect observed was the production of longer stubs at some I2s (Table 2). This phenotype of longer I2 stems and stubs, although not very dramatic, is consistent with a role of *PsHUP54* in regulating the period of time in which the I2 meristem stays active.

To connect the observed phenotypes in the VIGS plants with the causal gene, we studied the silencing of *PsHUP54*-VIGS in *PsHUP54*-VIGS plants, the plants with a more clear I2 phenotype. With that aim, we analyzed by RT-qPCR the expression level of the endogenous *PsHUP54* gene in *PsHUP54*-VIGS plants and compared it with its expression in *GUS*-VIGS control plants. As expected, the *PsHUP54*-VIGS plants analyzed showed a lower *PsHUP54* expression level than control *GUS*-VIGS plants (Supplementary Figure 7). In general, in these plants, the strength of the phenotype correlated with the level of *PsHUP54* expression (Supplementary Figure 7). These results indicate that in *PsHUP54*-VIGS plants, the expression of the *PsHUP54* gene was silenced, which might be the cause of their phenotype.

## Discussion

The recent development of genomic resources, such as high-quality transcriptome assemblies and full-genome sequences (Alves-Carvalho et al., 2015; Kreplak et al., 2019), represent an important step forward for molecular genetic studies in pea, one of the most studied model plants among legume crops. These new resources have been instrumental for this study aimed at understanding the control of I2 development.

Our experimental approach has been based on the idea that it would be possible to identify genes expressed in the I2 meristem (therefore, candidates to be regulated by VEG1) by comparing the transcriptomes of inflorescence apices from pea mutants with defects in I2 development. This experimental strategy has proved to be successful, and the comparison of inflorescence apex transcriptomes from wild-type pea, the *pim* mutant (enriched in I2 meristems), and *veg1* and *veg2* mutants (both without I2 meristems) (Taylor et al., 2002; Berbel et al., 2012; Sussmilch et al., 2015) has allowed us to identify a number of genes with preferential expression in the I2 meristem.

In our study, the selection of promising candidate genes from the list of genes differentially expressed (DEGs) between the different mutants has suffered from the still limited functional annotation of the pea genome, only relatively recently published (Kreplak et al., 2019). To overcome this limitation, we have used functional information on the homologs of the pea genes in *Medicago truncatula* and Arabidopsis. Since some Medicago databases are still not fully developed, ultimately our main source of information has been the Arabidopsis databases, even though Arabidopsis is phylogenetically not so closely related to pea, and the available knowledge for Arabidopsis genes is not ideal to make predictions about their pea homologs. Nevertheless, the Arabidopsis-based information, combined with expression studies and functional analysis by VIGS in pea, has allowed us to identify several interesting genes likely involved in the development of the I2.

Several candidate genes with an interesting expression pattern were identified. Thus, 8 out of 12 genes whose expression pattern was analyzed by RT-qPCR showed moderate to a high expression level in the inflorescence apex. Among them, the *PsDAO1* gene showed much higher expression in the inflorescence than in the vegetative apex. Moreover, the expression of *PsKMD2, PsLBD38*, and *PsTAR2* was also detected in the I2 meristem by *in situ* hybridization.

*PsTAR2* encodes a key enzyme involved in the initial steps of auxin synthesis, which belongs to a small gene family of at least two additional members (Tivendale et al., 2012). Interestingly, *PsTAR2-*specific expression in the I2 meristem of the inflorescence apex suggests that local auxin production in this domain could be important for the correct specification or development of the secondary inflorescence. However, when VIGS was used to study the function of *PsTAR2*, no evident phenotypic defect was observed, maybe because redundancy with other close homologs precluded the effective reduction of TAA/TAR activity (Tivendale et al., 2012; Bala et al., 2017). Likewise, in *PsLBD38*-VIGS plants, only a subtle phenotype was observed, where the flower pedicels were significantly shorter than in the *GUS*-VIGS control plants. This phenotype, together with the *in situ* hybridization data, which showed that *PsLBD38* is expressed in the floral meristem, may suggest a possible role of *PsLBD38* in the floral development in pea. The LBD family of plant-specific transcription factors is relatively large, with 43 members in Arabidopsis, and can be grouped into two classes. Functional studies have associated class I LBD genes from different species with general roles in lateral organ patterning and in auxin signal transduction (Xu et al., 2016), while for class II genes, to which *PsLBD38* belongs, functional information is still limited, although they appear to be involved in metabolic processes, such as anthocyanin synthesis in response to N availability (Rubin et al., 2009). Intriguingly, the potential function of *PsLBD38* in the control of floral pedicel length resembles more the described role of class I LBD genes in petiole development of leaves in legumes, expanding the evidence on the functional versatility of the family (Chen et al., 2012). Again, it is possible that redundancy masked the phenotypic effects of *PsLBD38* silencing, making it necessary to address this possibility in future studies.

In contrast, the *PsDAO1-*VIGS plant exhibited a dramatic phenotype. *PsDAO1*-VIGS plants were very small, with short internodes, small leaves, and short floral pedicels. *PsDAO1* is a homolog of the Arabidopsis *DAO1* (*DIOXIGENASE FOR AUXIN OXIDATION 1*) gene, which encodes an indole acetic acid (IAA) oxidase, the major contributor to IAA oxidation in Arabidopsis, whose activity is tightly coordinated with auxin biosynthesis and conjugation (Porco et al., 2016; Zhang et al., 2016). The VIGS-*PsDAO1* phenotype could possibly reflect an alteration in these VIGS plants of auxin homeostasis, a hormone with a key role in the regulation of plant growth. The dramatic reduction in organ size found in the pea VIGS-*PsDAO1* plants contrasts with the phenotype of Arabidopsis *dao1* mutants that, apparently depending on the growing conditions, show either a slight reduction in inflorescence stem and siliques (Porco et al., 2016) or moderate enlargement of rosette leaves and inflorescence stem (Zhang et al., 2016). However, it should be noted that in Arabidopsis, two closely related genes, *AtDAO1* and *AtDAO2*, are found in tandem in the genome (At1G14130 and At1G14120, respectively), and the double mutant has not been generated yet, so the full consequences of the lack of IAA oxidation have not been uncovered so far. Moreover, functional analyses of *DAO1* homologs in rice show a prominent role of these enzymes in reproductive development, where the mutants showed severe defects in anther dehiscence, pollen maturation, and flower aperture (Zhao et al., 2013). The *PsDAO1*-VIGS plants from this work show a different effect of potentially reduced auxin catabolic processes. It is clear, then, that more studies are required to better understand the full spectrum of IAA oxidation role in the development and how it is integrated into auxin signaling pathways.

Finally, despite a relatively low expression level in the inflorescence apex, *PsHUP54* expression seemed spatially restricted to the I2 meristem. The most prominent effect of silencing *PsHUP54* is a dramatic increase in plant growth, which affects most aerial organs, including plant height, leaf size, and pod length, with a subsequent increase in seed production, indicating that *PsHUP54* could function as a general repressor of growth. Regarding I2 activity, a conspicuous defect in *PsHUP54*-VIGS plants was that the stubs, the residual organs formed by the I2 meristems after producing the flowers (Benlloch et al., 2015), were usually much longer than in the control *GUS*-VIGS plants. Together with the specific expression of *PsHUP54* in the I2 meristem, this suggests that *PsHUP54* could be a target of VEG1 that promotes I2 meristem termination, so that in the *PsHUP54-*VIGS plants the I2 meristems stay active for longer. Accordingly, in the *PsHUP54*-VIGS plants, a higher number of flowers per node was observed in one plant, which supports this hypothesis.

The Arabidopsis *HUP54* gene belongs to a small gene family with homologs present in all plant groups, which contain conserved YGL and LDRD motifs (Cheng et al., 2017). Despite the high level of conservation, especially in angiosperms, where homologs with a percentage of identity higher than 60% can be found even in the most basal clades, limited functional information based on mutant phenotypes is available for these proteins, and their molecular function is still basically unknown. In Arabidopsis, *AtHUP54* appears to be involved in hypoxia tolerance (Mustroph et al., 2010) and plant cell wall remodeling as a target of the SHINE transcription factors in a GA-dependent manner (Shi et al., 2011). The possible link of these putative functions with the control of plant growth in pea remains to be explored in detail.

It seems noticeable that the conspicuous phenotype of *PsHUP54*-VIGS plants, which were notably larger, featured longer pods with up to double the number of seeds, with no concomitant decrease in seed weight, has not apparently been identified in mutant screenings in pea or other legumes. A possible explanation is that the *PsCam027351* gene, present in the pea genome, with a high level of similarity to *PsHUP54* (77% nucleotide identity), is functionally redundant to *PsHUP54*. Since the *PsHUP54-*VIGS construct might be silencing both genes, the phenotype of the *PsHUP54-*VIGS could be equivalent to that of a double *PsHUP54 PsCam027351* mutant, something that will have to be considered if these genes are to be exploited in breeding programs. Notably, our work has revealed a novel role for a protein of unknown function in growth control, which looks a promising tool to improve yield in pea and possibly also in other legumes where highly conserved homologs of *PsHUP54* are present (Supplementary Figure 8).

In summary, this study presents a successful strategy to identify genes with expression in the I2 meristem of the pea inflorescence, likely controlling different aspects of inflorescence architecture in legumes. Although more detailed functional analyses should be carried out to elucidate the precise functions of these genes, our approach has already served as a proof of concept to validate the use of the new genomic tools available for pea and to identify at least one novel gene that is a potential target for breeding programs.

## Data availability statement

The datasets presented in this study can be found in online repositories. The names of the repository/repositories and accession number(s) can be found at: NCBI GEO, accession no: GSE188301.

## Author contributions

MS-P, RB, JW, and FM designed the experiments and interpreted the data. MS-P performed the experiments. MS-P, VH, RB, and FM analyzed the transcriptomic data. MS-P, RB, and FM wrote the manuscript with the input of all authors. All authors approved the submitted version.

## Funding

This work was supported by the Spanish Ministerio de Ciencia Innovación y Universidades and FEDER and by the Generalitat Valenciana (grants BIO2015-64307-R and PGC2018-099232-B-I00 to FM, BIO2015-73491-JIN to RB and PROMETEO/2019-004 to FM and RB) and by the Australian Research Council (grants DP120101241 and DP160100793 to JW). MS-P was supported by a contract for personal predoctoral en formación from the Generalitat Valenciana. We acknowledge the support of the publication fee by the CSIC Open Access Publication Support Initiative through its Unit of Information Resources for Research (URICI).

## Conflict of interest

The authors declare that the research was conducted in the absence of any commercial or financial relationships that could be construed as a potential conflict of interest.

## Publisher's note

All claims expressed in this article are solely those of the authors and do not necessarily represent those of their affiliated organizations, or those of the publisher, the editors and the reviewers. Any product that may be evaluated in this article, or claim that may be made by its manufacturer, is not guaranteed or endorsed by the publisher.
