# Supplementary material for: Identification and characterization of putative targets of VEGETATIVE1/FULc, a key regulator of development of the compound inflorescence in pea and related legumes
Source: Front Plant Sci. 2022 Sep 21;13:765095. doi: 10.3389/fpls.2022.765095 (PMC9533771; doi:10.3389/fpls.2022.765095)
Supplement: Supplementary file 3 [file Data_Sheet_1.pdf]

Supplementary Material

1.1 Supplementary Figures

A

sample	total reads	mapped reads
WT 1	19947426	18027806
WT 2	22907817	20577332
WT 3	23282606	20985906
veg1 1	18820513	20135449
veg1 2	21665464	20678503
veg1 3	19379188	17197199
veg2 1	18292370	16871067
veg2 2	22244206	19167117
veg2 3	20033619	17488509
pim 1	22277701	16478666
pim 2	22700243	20303380
pim 3	18748305	17826342

B

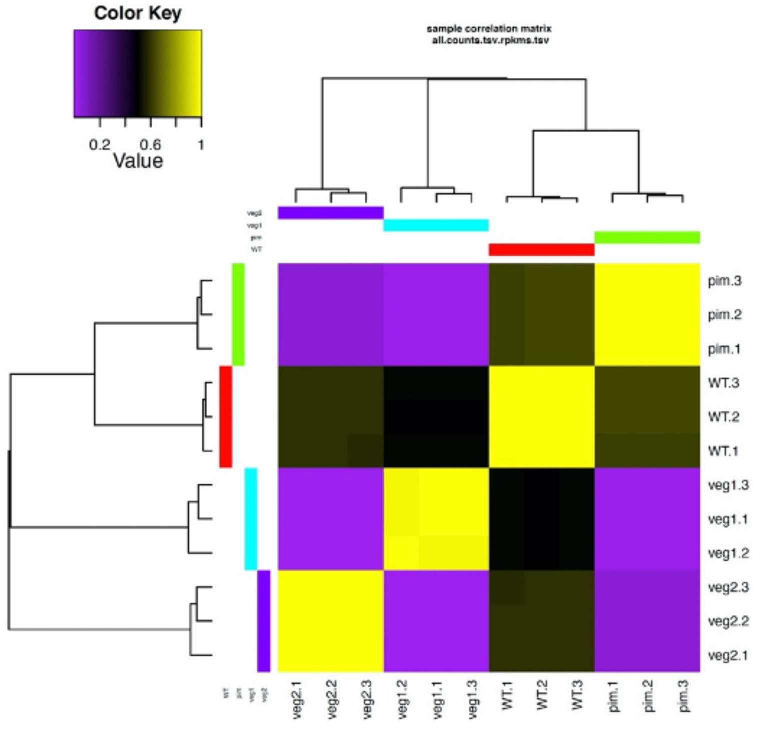

Supplementary Figure 1 Quality analysis of the RNAseq. (A) Number of reads. Mapped reads refer to sequences that can be aligned to the pea reference genome. **(B)** Correlation matrix. Color code indicates similarity between samples.

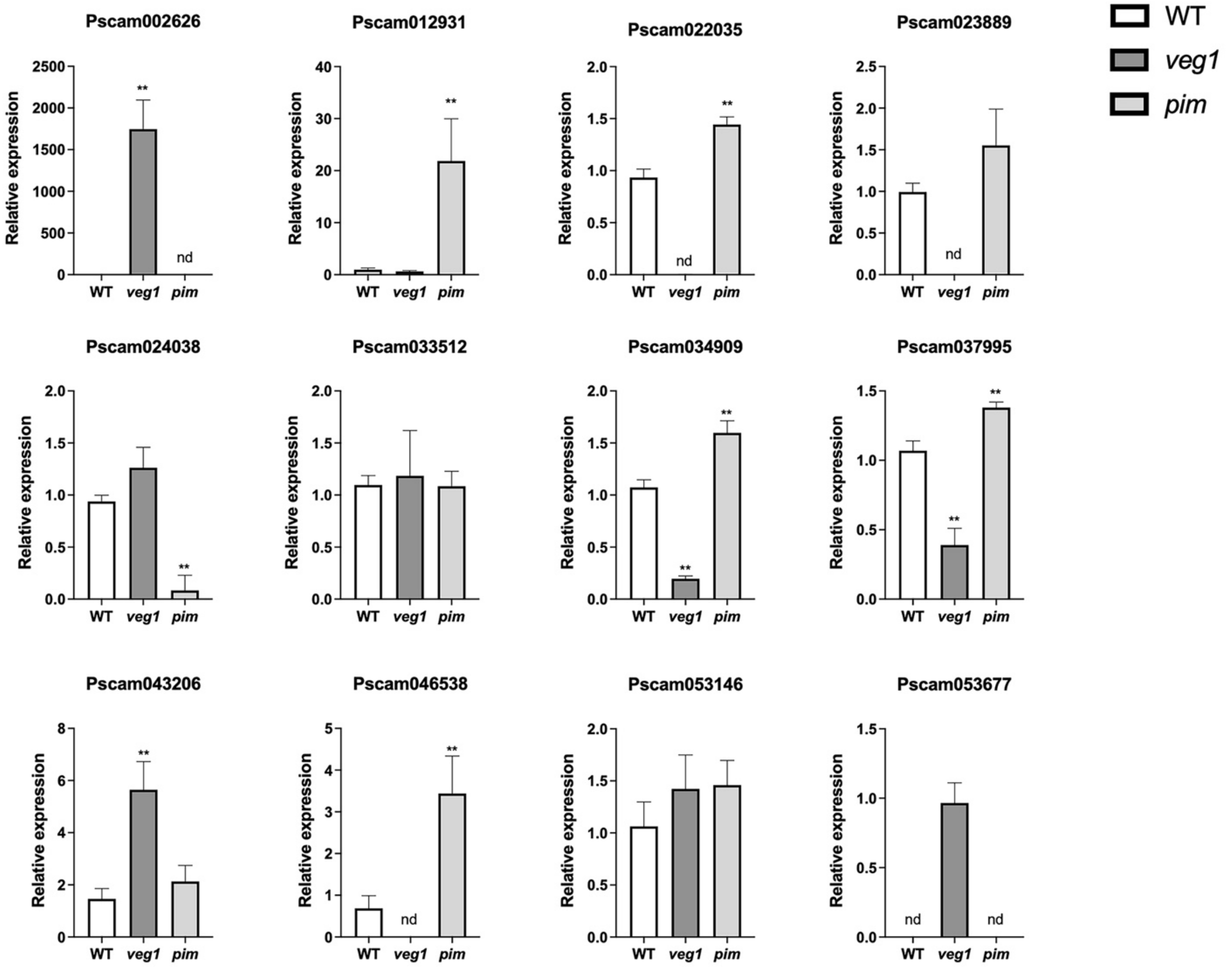

Supplementary Figure 2. Validation of RNAseq data by RT-qPCR analysis. RT-qPCR expression analysis, in wild type, *veg1*, and *pim* inflorescence apices, of 12 genes randomly chosen among those showing opposite expression between WT/*veg1* and WT/*pim*. nd: not detected.

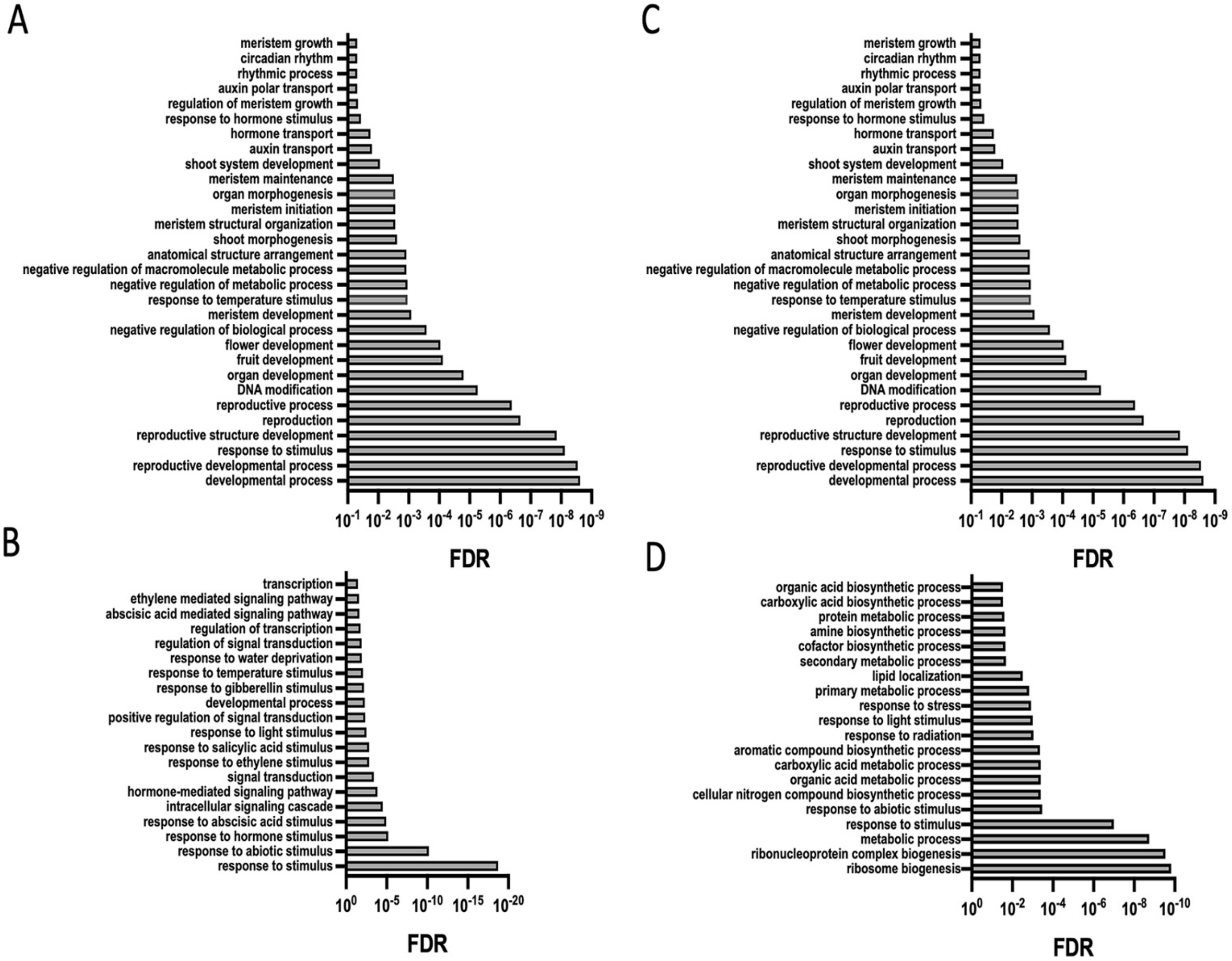

Supplementary Figure 3. Gene ontology term (biological processes) enrichment among differential expressed genes in WT/*pim and* WT/*veg2* transcriptomes. (**A)** Selected GO terms returned from the analysis of genes up-regulated in *pim*. **(B)** Selected GO terms returned from the analysis of genes down-regulated in *pim*. **(C)** Selected GO terms returned from the analysis of genes up-regulated in *veg2*. **(D)** Selected GO terms returned from the analysis of genes down-regulated in *veg2*. Terms were selected according to their relation to previously described PIM or VEG2 function and/or potential mechanisms of action. All depicted terms were overrepresented (False Discovery Rate (FDR) < 0.05). A complete list of all enriched GO terms for each analysis is detailed in Supplementary Table 3.

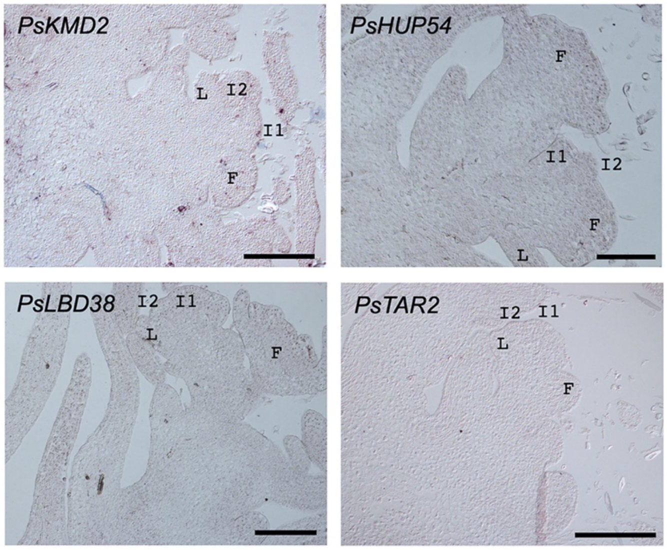

Supplementary Figure 4. Negative controls (sections hybridized with sense probes) for *in situ* hybridization experiments in Figure 6. L, leaf primordium; I1, primary inflorescence meristem; I2, secondary inflorescence meristem; F, floral meristem/primordium Scale bars: 200 µm

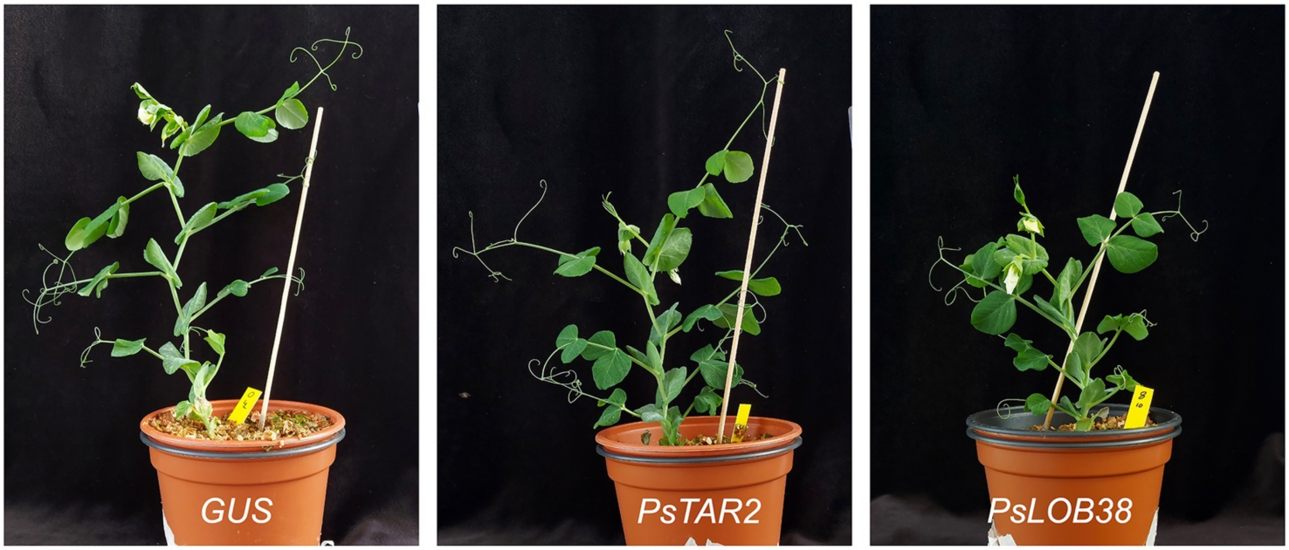

Supplementary Figure 5. Phenotype of pea VIGS plants for genes *PsTAR2* and *PsLOB38* Representative six-week-old *GUS*-VIGS (control), *PsTAR2*-VIGS and *PsLOB38*-VIGS plants.

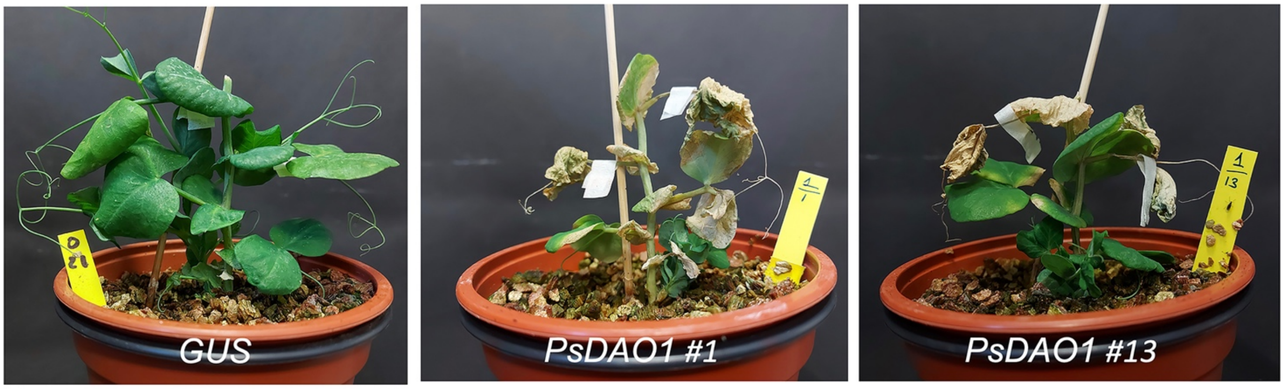

Supplementary Figure 6. Morphological alteration of plants infiltrated with the VIGS- *PsDAO1* construct. Pea plants three weeks after infiltration with the *GUS*-VIGS or *PsDAO1*-VIGS constructs.

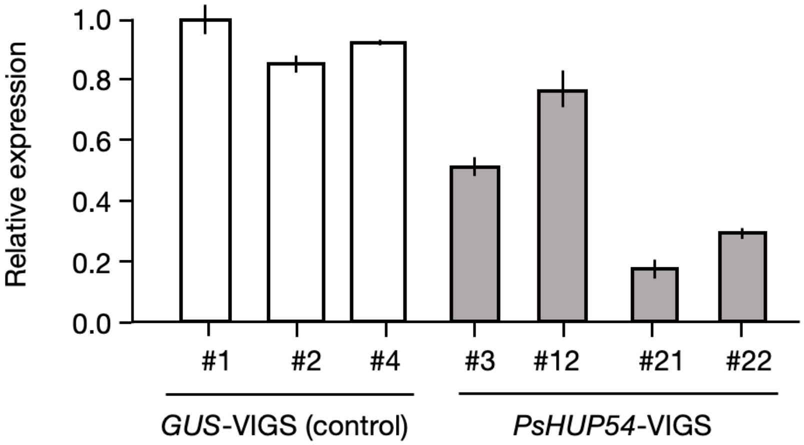

Supplementary Figure 7. Expression of *PsHUP54* gene in *PsHUP54*-VIGS plants. Relative mRNA levels were determined by RT-qPCR. Samples were leaflets of fully expanded leaves at equivalent nodes. While *GUS*-VIGS plants exhibited a wild-type phenotype, *PsHUP54*-VIGS plants number #3 and #12 exhibited a weak phenotype and plants number #21 and #22 a moderate-strong phenotype. Error bars correspond to standard deviation of three technical replicates.

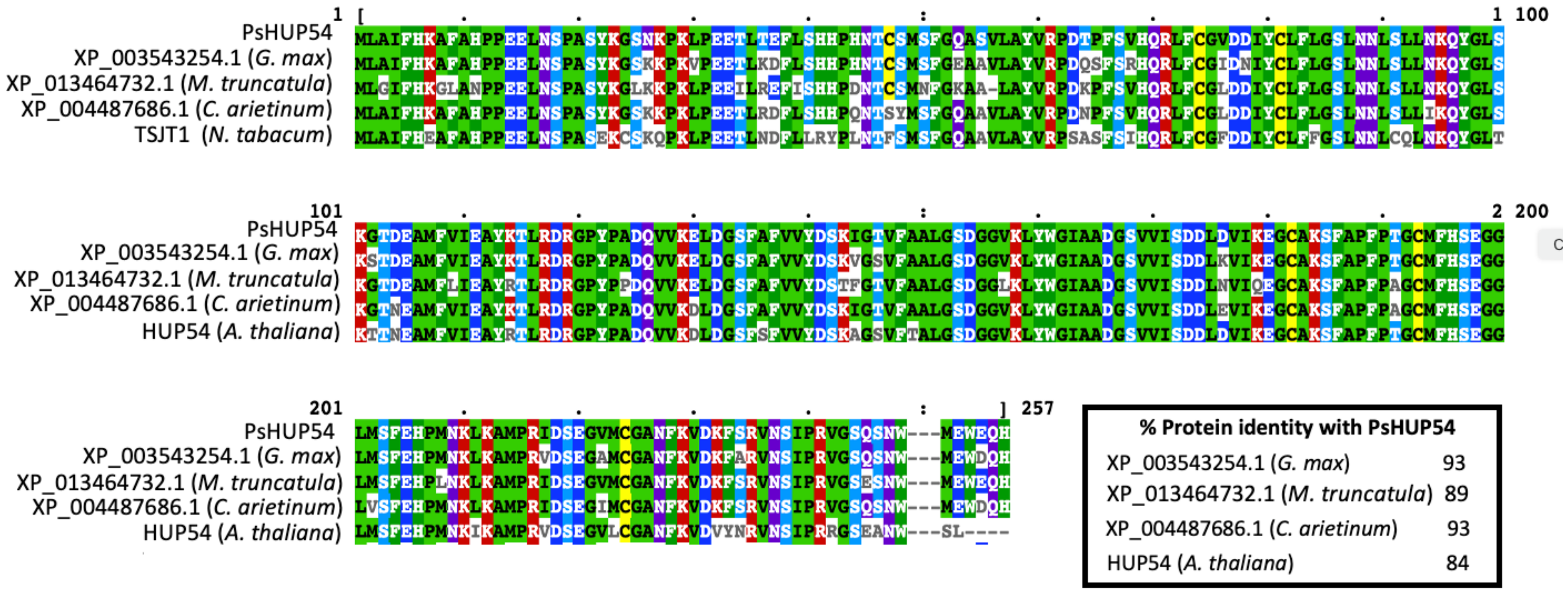

Supplementary Figure 8. Sequence alignment of PsHUP54 with homologues from *Glycine max* (XP_003543254.1), *Medicago truncatula* (XP_013464732.1), *Cicer arietinum* (XP_004487686.1) and *Arabidopsis thaliana*. The percentage of protein identity for each homologue is shown in the square.

1.2 Supplementary Tables

Supplementary Table 1. Primers used in this work

Primer name	Sequence
**RT-qPCR primers for detecting floral transition (Fig 1)**	
**VEG1 F**	CACTCAGTGGTGCTATACAAG
**VEG R**	GCATCCATGCAGGAATAAGA
**PIM F**	TGCAGCTGAGCAGCAGGTA
**PIM R**	TAGTAAGTAATTTGGATTGACTCCATG
**RT-qPCR primers for reference control gene**	
**ACTIN11 (PEAc14) F**	AGGTGCTGTACCAACCATCCA
**ACTIN11 (PEAc14) F**	CGTGAATTCCTGCTGCTTCC
**RT-qPCR primers for RNAseq validation (Supplementary Fig 1)**	
**Pscam002626 F**	AGTTAAATCCGCTGCTGCTG
**Pscam002626 R**	TGGGAATTTCGGCTTGGATG
**PsCam012931 F**	TTCGGCAGAAGATGAGGATGAG
**PsCam012931 R**	TGCCACTGCAACAAAAGCTG
**PsCam022035 F**	ATAACCACGGACATGCCTTG
**PsCam022035 R**	AATCATCGTCGCCTACCAAC
**PsCam023889 F**	AACAGGGCCATGGTCAATTG
**PsCam023889 R**	TTTCCAAACGCAAGCCAAGC
**PsCam024038 F**	AACGGAGATGAAGGCGTTTG
**PsCam024038 R**	ACCACACTCCAATCGTCAAC
**PsCam033512 F**	GGCGTGATTGCAAAATCGAG
**PsCam033512 R**	TTGCCACTTCAATGCGTTGC
**PsCam034909 F**	AACCTGGTTTGCACCTTCAC
**PsCam034909 R**	TGATTTTGGGTGCAGCACTG
**Pscam037995 F**	AGGTTTACCATGTCGGGTCTTC
**Pscam037995 R**	TCCATTCTACCAGGACGCAAC
**PsCam043206 F**	TGTGTGGCTGCATTTGCTTC
**PsCam043206 R**	ACATGGTGTGGTCCAAACTC

**PsCam046538 F**	AACCACTCATTCAGCTGTGC
**PsCam046538 R**	TCCCAAGTAGTTCAAGCTCAGG
**PsCam053146 F**	TGTGATTCCTGCCTTTGCAC
**PsCam053146 F**	GTGGAAGAGACCTTGTGCAAAG
**PsCam053677 F**	AATCTGGCCGTTGAATCTGC
**PsCam053677 R**	GGGCTTACACCAACAGACAAAC
**RT-qPCR primers for expression of candidate genes (Fig 5)**	
**PsCam001113 F**	AACTCAGCCATGCAAAGACG
**PsCam001113 R**	AGTTGTTCAGTCGTGTGAGC
**PsCam016925 F**	TTTTTCAAGGGGTGGTGGTG
**PsCam016925 R**	ATGTCGTAACCTCCACCGTATG
**PsCam037476 F**	TTTTGGGGAGCCTTCAAACC
**PsCam037476 R**	AAAGCAACCACGTGAACAGC
**PsCam039164 F**	TCGTGTGCAATGCAAGGAAG
**PsCam039164 R**	TTGGAGCCTCAACATTTCCC
**PsCam042718 F**	TGCAGCTGCTGTAATGCATG
**PsCam042718 R**	TCCGTGACAACAGTTTTGGC
**PsCam043276 F**	TTCCGAACGAACATGTGGAG
**PsCam043276 R**	TGTCCACCGGCAACAAAAAC
**PsCam043354 F**	TGCAGCTGATGGATCTGTTG
**PsCam043354 R**	GCAAAGGATTTAGCGCAACC
**PsCam044132 F**	GCCTAGTTGCTGCAATTGTG
**PsCam044132 R**	AACTTGCCTCGAAGAACCAC
**PsCam044818 F**	TTACATTTGCGGTGCGTGTG
**PsCam044818 R**	TCTTGACACTTGGCATTGGC
**PsCam046067 R**	ATTTGCACCCCCAAACCAAG
**PsCam046067 R**	TTCCCCTGTTCACTGCAAAC
**PsCam047398 F**	AATGGTGGTGCAGTTCCATG
**PsCam047398 R**	ACAACAACGCGGAGAAACAG
**PsCam048048 F**	AAGCCCGCACAATCAAAAGG
**PsCam048048 R**	TGACATTTCGGCAGCTTCAG
**PsCam050808 F**	ACGCAAGCGATTGAGTGAAG
**PsCam050808 R**	AACTGTTGGTCAACGCGTTC
**PsCam057706 F**	AGGTCGCCACATTGTTGTTG
**PsCam057706 F**	TGGCAGAAACAACGCTGATG

**PCR primers for probes for *in situ* hybridization (Fig 6)**	
**PsCam039164 F**	ATGAAAATGTTGGTGGTCTTGAAAT
**PsCam039164 R**	TTAAACCAAGCGTAATAACTCGAGA
**PsCam043276 F**	TTGGTGACTGGGTCCGAATACGAAG
**PsCam043276 R**	AAAGTTCACTGCGAAACCATACATG
**PsCam043354 F**	ATCCAATGAACAAGTTGAAAGCAAT
**PsCam043354 R**	AAACACTTGAATATATTGTGTTACT
**PsCam050808 F**	ACAGTTACCGGAGGGACGTTTG
**PsCam050808 R**	TCCTCACCGACTAACCTTCATCTTT
**PsCam057706 F**	ATATTCTGAAACCAAGTTGTTTTCT
**PsCam057706 R**	TTTTTGAATAATCCGATTATACCCT
**Primers for VIGS constructs (Fig 7)**	
**PsDAO1-VIGS F**	TCCATGGATGAAAATGTTGGTGGTCTTGAAAT [a] (NcoI)
**PsDAO1-VIGS R**	TGAATTCTTAAACCAAGCGTAATAACTCGAGA (EcoRI)
**PsHUT54-VIGS F**	TCCATGGATCCAATGAACAAGTTGAAAGCAAT (NcoI)
**PsHUT54- VIGS R**	TGAATTCAAACACTTGAATATATTGTGTTACT (EcoRI)
**PsLOB38- VIGS F**	TCCATGGACAGTTACCGGAGGGACGTTTG (NcoI)
**PsLOB38- VIGS R**	TCTGCAGTCCTCACCGACTAACCTTCATCTTT (EcoRI)
**PsTAR2-VIGS F**	TCCATGGATATTCTGAAACCAAGTTGTTTTCT (NcoI)
**PsTAR2-VIGS R**	TGAATTCTTTTTGAATAATCCGATTATACCCT (EcoRI)

[a]Restriction sites in the primers are underlined

Supplementary table 5. Expression in apices of the wild type and inflorescence mutants of differentially expressed genes selected in the RNAseq

Gene	WT.1	WT.2	WT.3	*pim*.1	*pim*.2	*pim*.3	*veg*.1	*veg1*.2	*veg1*.3	*veg2*.1	*veg2*.2	*veg2*.3
**PsCam057706 (PsTAR2)**	29,12923581	24,81263069	26,60744208	21,44768739	20,59579969	20,2895286	29,21786753	33,73239341	33,69758881	23,49937984	22,8840393	26,08290132
**PsCam050808 (PsLBD38)**	16,18715214	12,6491135	17,67164926	21,86352428	23,16896608	24,20932444	11,35551533	12,17574679	9,984105362	17,35162065	14,78276487	19,56614806
**PsCam043354 (PsHUP54)**	26,20173012	29,63940199	30,90873789	49,90875496	37,62831223	46,77513186	20,78234566	18,67221005	16,72519771	24,85425326	16,49832088	16,12416602
**PsCam039164 (PsDAO1)**	2,70654693	2,000426903	2,122931601	3,42199887	4,185182876	4,194650284	0,450685502	0,352716752	0,435020217	0,359457201	0,166687939	0,14286344
**PsCam043276 (PsKMD2)**	9,662992204	10,60429628	9,167478216	16,07674338	13,11735116	18,01739915	7,003917346	7,888447503	5,575192488	18,42194398	11,3541507	10,6395723
**PsCam048048**	15,51363341	14,60100607	13,94147845	16,47652748	19,70339514	19,98112694	13,07017755	11,38770934	11,59857638	12,90297957	10,98403012	9,149894909
**PsCam047398**	5,48793613	6,961712573	5,652790082	4,388751006	2,412713488	2,611625056	7,459588995	12,70350796	10,3267499	2,214328544	1,796954564	2,053490792
**PsCam046067**	4,028163505	4,423083766	4,022151546	1,89783506	2,85518863	2,564615666	5,734134511	8,155467115	10,72904134	9,815271751	9,837220951	10,36072744
**PsCam044818**	20,93567411	20,65679786	19,57989002	34,86220136	30,31321528	37,55418114	18,02759311	16,69139744	16,9618036	19,39895171	19,11647614	19,08603372
**PsCam044132**	175,5336548	196,7896795	190,2434835	173,9862337	164,9796992	150,2084323	314,9897582	278,2693885	252,8411005	248,059657	239,7031382	264,4054791
**PsCam042718**	27,66738159	27,88292064	24,36414012	23,74999511	22,60810036	22,91771316	33,64177881	36,67082348	28,2192853	35,75338023	22,67015266	30,56244972
**PsCam037476**	10,00170497	9,28527562	9,267733906	11,27010521	12,34427738	13,58516263	0	0	0	9,794468083	9,259394809	8,864554364
**PsCam016925**	104,1551974	89,86639374	94,19153245	106,0547118	113,1279995	119,3744001	66,70270876	60,64841213	68,92411485	101,2420372	97,53062692	94,1322933
**PsCam001113**	28,50408007	30,78969324	28,85386523	50,48271504	37,99644387	48,29140828	23,18621008	26,53860808	20,91695905	42,61479519	29,20211206	29,72440791

Data correspond to the RPKN value for each gene, for the 14 selected DEGs, in samples of inflorescence apices from the wild type and the three mutant genotypes.

Supplementary Table 6. Characterization of morphological alterations in *PsLOB38*-VIGS and *PsTAR2*-VIGS plants

VIGS construct	Parameters			
	Internode length[b] (cm)	Leaflet number[c]	I2 length (cm)	Floral pedicel length (cm)
**GUS**	2.07 ± 0.38	3.30 ± 0.98	7.78 ± 5.50	4.83 ± 1.29
**PsTAR2**	1.75 ± 0.40	3.22 ± 0.81	5.47 ± 2.22	4.10 ± 1.02
**PsLOB38**	1.91 ± 0.33	3.68 ± 0.75	5.10 ± 2.47	**3.30 ± 2.51** [a.]

Values correspond to mean ± standard deviation.

[a] The data in bold correspond to values with statistically significant variation respect the values of control *GUS*-VIGS plants. For statistical analysis, one-way ANOVA test with Bonferroni and Holm inference test were used.

[b] Values correspond to the average of the internodes of the stem before the first reproductive node

[c] Leaflet number correspond to the leaves at the first reproductive node and at the previous one.
